# Cardiovascular disease incidence after internal mammary chain irradiation and anthracycline-based chemotherapy for breast cancer

**DOI:** 10.1038/s41416-018-0159-x

**Published:** 2018-08-01

**Authors:** Naomi B. Boekel, Judy N. Jacobse, Michael Schaapveld, Maartje J. Hooning, Jourik A. Gietema, Frances K. Duane, Carolyn W. Taylor, Sarah C. Darby, Michael Hauptmann, Caroline M. Seynaeve, Margreet H. A. Baaijens, Gabe S. Sonke, Emiel J. T. Rutgers, Nicola S. Russell, Berthe M. P. Aleman, Flora E. van Leeuwen

**Affiliations:** 1 Epidemiology, Netherlands Cancer Institute, Plesmanlaan 121, 1066 CX Amsterdam, The Netherlands; 2 Department of Medical Oncology, Erasmus MC - Cancer Institute, Groene Hilledijk 301, 3075 EA Rotterdam, The Netherlands; 3 Medical Oncology, University Medical Center Groningen, Hanzeplein 1, 9213 GZ Groningen, The Netherlands; 4 Nuffield Department of Population Health, University of Oxford, Old Road Campus, Oxford, OX3 7LF UK; 5 Medical Research Council Population Health Research Unit, Nuffield Department of Population Health, University of Oxford, Old Road Campus, Oxford, OX3 7LF UK; 6 Radiation Oncology, Erasmus MC - Cancer Institute, Groene Hilledijk 301, 3075 EA Rotterdam, The Netherlands; 7 Medical Oncology, Netherlands Cancer Institute, Plesmanlaan 121, 1066 CX Amsterdam, The Netherlands; 8 Surgery, Netherlands Cancer Institute, Plesmanlaan 121, 1066 CX Amsterdam, The Netherlands; 9 Radiation Oncology, Netherlands Cancer Institute, Plesmanlaan 121, 1066 CX Amsterdam, The Netherlands

**Keywords:** Breast cancer, Epidemiology

## Abstract

**Background:**

Improved breast cancer (BC) survival and evidence showing beneficial effects of internal mammary chain (IMC) irradiation underscore the importance of studying late cardiovascular effects of BC treatment.

**Methods:**

We assessed cardiovascular disease (CVD) incidence in 14,645 Dutch BC patients aged <62 years, treated during 1970–2009. Analyses included proportional hazards models and general population comparisons.

**Results:**

CVD rate-ratio for left-versus-right breast irradiation without IMC was 1.11 (95% CI 0.93–1.32). Compared to right-sided breast irradiation only, IMC irradiation (interquartile range mean heart doses 9–17 Gy) was associated with increases in CVD rate overall, ischaemic heart disease (IHD), heart failure (HF) and valvular heart disease (hazard ratios (HRs): 1.6–2.4). IHD risk remained increased until at least 20 years after treatment. Anthracycline-based chemotherapy was associated with an increased HF rate (HR = 4.18, 95% CI 3.07–5.69), emerging <5 years and remaining increased at least 10–15 years after treatment. IMC irradiation combined with anthracycline-based chemotherapy was associated with substantially increased HF rate (HR = 9.23 95% CI 6.01–14.18), compared to neither IMC irradiation nor anthracycline-based chemotherapy.

**Conclusions:**

Women treated with anthracycline-based chemotherapy and IMC irradiation (in an older era) with considerable mean heart dose exposure have substantially increased incidence of several CVDs. Screening may be appropriate for some BC patient groups.

## Introduction

Breast cancer (BC) survival has improved substantially in recent decades due to earlier diagnosis and treatment advances.^1–5^ At present, both radiation therapy (RT) and anthracycline-based chemotherapy are commonly used. They cure many women of their cancer but both treatments have been associated with increased risks of cardiovascular disease (CVD).^6,7^ Radiation-related CVDs include ischaemic heart disease (IHD) and valvular heart disease (VHD), with evidence for dose-dependency.^8–10^ Previously, RT-related CVDs were thought not to emerge until 10 years after exposure.^11–15^ Recently, however, increased risks have been observed within 5 years of exposure.^8,16^ Anthracycline-based chemotherapy is associated with an increased, dose-dependent risk of cardiomyopathy (CMP) and heart failure (HF).^17–19^ However, the reported cumulative HF incidence after anthracycline-based chemotherapy varies.^20–23^

Since the 1970s, thousands of women in the Netherlands have been treated with internal mammary chain (IMC) irradiation using techniques that deliver substantial radiation doses to the heart. Since the 1990s, many women in the Netherlands have also received anthracycline-based chemotherapy. The absolute heart disease risks for women treated in the past are currently unclear, and it is not known which women might benefit from surveillance for heart disease.

Recent randomised trials have reported a BC-specific survival benefit after nodal irradiation, including IMC irradiation.^24,25^ This has re-opened the debate on the role of IMC irradiation in BC treatment.^26^ Women given IMC radiotherapy today may still receive around 8 Gy,^27–30^ but some cancer centres achieve much lower heart doses.^28,29,31^ Many of these women also receive anthracycline-based chemotherapy. Identifying interactions between RT and anthracycline-based chemotherapy or established cardiovascular risk factors^8,11,32^ is therefore relevant to women treated today.

Here we report the separate and combined effects of various radiation fields, chemotherapy types and established cardiovascular risk factors on the long-term risks of IHD, VHD and HF in a large cohort of BC patients aged <62 years at diagnosis.

## Methods

### Data collection procedures

Female BC patients (stages I–IIIA or ductal carcinoma in situ [DCIS]) were selected from the hospital-based registries of the Netherlands Cancer Institute, Amsterdam or the Erasmus MC - Cancer Institute, Rotterdam, the Netherlands. All patients were diagnosed during 1970–2009 and before the age of 62 years. Data collection procedures have been published previously.^11^ In brief, patient and tumour characteristics, BC treatments (also locoregional recurrences and subsequent BCs) and CVD events were collected from registries and patient records. Patients were scored positive for hypertension, diabetes mellitus or hypercholesterolaemia if they received treatment for these conditions. Supplementary methods I shows detailed data collection procedures and patient eligibility criteria.

To complete information on CVD incidence, cardiovascular risk factors and causes of death, questionnaires were sent to general practitioners (GPs)^i^ and, if applicable, cardiologists of all patients. (^i^In the Netherlands, all residents are expected to have a primary care physician. Medical correspondence from attending physicians is sent to the primary care physician. Such records are preserved by the primary care physicians throughout a patient’s life and for at least 15 years after a patient’s death). Date of death was acquired through the population-based municipal personal records database.

In the current study, women treated with trastuzumab or taxanes (with or without anthracycline-based chemotherapy) for their primary BC (*n* = 979) were excluded, since follow-up was short and the numbers of events were too small to examine the effects of these treatments on CVD risks. The total analytic cohort comprised 14,645 patients.

### Treatment

A detailed description of the treatment modalities used in our cohort from 1970 to 1986 has been published previously.^11^ During the 1970s, standard treatment for stage I–IIIa BCs consisted of mastectomy, with/without RT. In 1975, CMF (cyclophosphamide, methotrexate and fluorouracil) chemotherapy was introduced for premenopausal lymph node-positive patients. Breast-conserving surgery followed by whole breast irradiation was introduced in 1980. For women who underwent mastectomy, chest wall irradiation was indicated following incomplete resection or for extensive locoregional tumours. Regional nodal irradiation, including IMC irradiation, was used for women with positive axillary nodes and, in some cases, medial tumours. From the 1990s, anthracycline-based chemotherapy was used for most premenopausal, and later also for postmenopausal, lymph-node positive patients and for lymph-node negative patients with unfavourable tumour characteristics. Most common anthracycline dose was four times 60 mg/m^2^ (doxorubicin equivalent) during the study period. DCIS was treated with either wide local excision followed by whole-breast RT or with mastectomy.

In previous decades, IMC irradiation usually consisted of direct photon beams, sometimes combined with electron beams, giving a total target dose of 36–54 Gy in 12–26 fractions. In the most recent treatment period, IMC irradiation consisting of a combination of oblique photon and electron beams giving a total target dose of 50 Gy (25 fractions) resulting in lower exposure of the heart was introduced.^33^ Chest wall irradiation usually consisted of a direct electron beam giving a total target dose of 35–46 Gy (15–23 fractions). Whole breast irradiation usually consisted of tangential photon beams giving a total target dose of 44–52 Gy (22–26 fractions); most women also received a boost dose to the tumour bed.

### Dosimetry

Dosimetry was performed to provide an indication of the typical level of cardiac exposure for women who received RT to different regions, according to laterality and IMC irradiation, during different time periods. Detailed information on the RT received was available for a sample of 683 women in the study cohort. Over 90% of these women were treated before the era of RT computed tomographic (CT) planning. Typical mean heart doses were estimated by reconstructing 44 different regimens on a “typical CT scan” (Supplementary methods II: Dosimetry). Dose distributions were generated for cobalt, electron and megavoltage beams using modern 3-dimensional CT treatment planning (Varian EclipseTM Treatment Planning System [TPS] version 10.0.39 [Varian Medical Systems, Palo Alto, USA]) and for orthovoltage fields using manual planning. A typical mean heart dose was allocated to each woman according to her regimen and total dose. Women were then categorised according to laterality and whether they received IMC irradiation. Within these categories, the typical doses were averaged. Given the large total number of women in the cohort, individual dosimetry was not undertaken and therefore no dose–response analyses have been performed.

### Statistical analysis

BC treatments received throughout follow-up (including treatment for contralateral BCs and locoregional recurrences) were classified time-varyingly. Chemotherapy regimens were categorised as CMF-like or anthracycline-based regimens. Differences in the likely radiation exposure of the heart were accounted for by considering laterality and radiation fields (breast, chest wall, IMC).

Because collection of CVD incidence for patients treated during 1970–1986 was restricted to 10-year survivors,^11^ time-at-risk started 10 years after BC diagnosis for patients diagnosed ≤1986 and 1 year after BC diagnosis for patients diagnosed >1986. Time-at-risk ended at date of event of interest, death, emigration, distant metastasis or date of last information, whichever came first.

#### General population comparisons

The incidence rate of myocardial infarction (MI) and HF (comprising congestive HF and CMP) in the cohort was compared with age-, sex- and calendar period-specific CVD incidence rates for the Dutch population.^34,35^ No comparable reference rates were available for VHD and angina pectoris (AP). We calculated standardised incidence ratios (SIRs) and absolute excess risks and estimated 95% confidence intervals (CIs).^36^

#### Within-cohort comparisons

We assessed the association between treatments and CVD risk using proportional hazard models. A cardiovascular event was defined as a CVD diagnosis or death due to CVD. We estimated risks for any CVD (ICD-10 I20–52) and separately for IHD (MI and AP), VHD and HF. When analysing a specific CVD, the presence of any other CVD was treated as a time-dependent covariate. Additionally, age at BC, CVD history, risk factors at BC diagnosis (dichotomised into yes/no) and smoking were included in the models as main effects. Treatment-specific cumulative CVD incidence was estimated in patients above and below 50 years at BC diagnosis (to avoid mixing different age/treatment distributions), in the presence of death from causes other than CVD as a competing risk.^37^ Model assumptions were verified using residual-based methods. Because the proportional hazard assumption did not hold for the IHD rate after IMC and chest wall irradiation, analyses are presented separately for <10 and ≥10 years after treatment.

We evaluated whether the observed data were consistent with an additive or a multiplicative model for the joint effect of two risk factors *A* and *B* by likelihood ratio tests of *γ* = 0 in models HR(*A*,*B*) = 1 + *β*_1_*A* + *β*_2_*B* + *γA*×*B* and HR(*A*,*B*) = exp(*α* + *β*_1_*A* + *β*_2_*B* + *γA*×*B*).^38^ Analyses were performed using Stata/SE 13.0 (StataCorp LP, College Station, TX) and EPICURE 1.8 (Hiro Soft International Inc, Seattle, WA). The study was approved by the review board of the Netherlands Cancer Institute.

## Results

The median follow-up duration of our cohort (*n* = 14,645) was 14 years, with 3486 patients followed ≥20 years. Median age at BC diagnosis was 47 years. Eighty six percent of patients received RT, of whom 36% had IMC irradiation. One third of the patients received chemotherapy (58% anthracycline-based). Few patients were treated for cardiovascular risk factors at BC diagnosis (4.6%), but >20% were current or past smokers (Table 1). A statistically significant but small difference in CVD history was observed between left- and right-sided BC patients (left-sided: 3.6%, right-sided: 3.0%). Other characteristics, including treatments, did not differ significantly by laterality (data not shown). BC treatment (including the receipt of IMC irradiation and anthracycline-based chemotherapy) was not associated with socioeconomic status, cardiovascular history at BC diagnosis or cardiovascular risk factors. (Supplementary table 7)

**Table 1:** Characteristics of hospital-based cohort of 14,645 breast cancer patients by year of breast cancer diagnosis

			Year of breast cancer diagnosis					
	Total		1970–1986		1987–1999		2000–2009	
Characteristic	No.	%	No.	%	No.	%	No.	%
Total no. of patients	14,645	100	3571	100	6626	100	4448	100
*Age at diagnosis (years)*								
Median (IQR)	47 (42–52)		47 (42–53)		46 (41–50)		51 (45–56)	
<35 years^a^	1010	6.9	236	6.6	562	8.5	212	4.8
35–40 years	1568	10.7	433	12.1	813	12.3	322	.2
40–49 years	6586	45.0	1600	44.8	3486	52.6	1500	33.7
50–61 years	5481	37.4	1302	36.5	1765	26.6	2414	54.3
*Stage*								
Ductal carcinoma in situ	929	6.3	40	1.1	318	4.8	571	12.8
I	4436	30.3	327	9.2	2168	32.7	1941	43.6
II	5251	35.9	433	12.1	3427	51.7	1391	31.3
IIIa	497	24.1	4	0.1	256	3.9	308	5.3
Unknown	3532	3.4	2767	77.5	457	6.9	237	6.9
*Type of surgery* ^b^								
Mastectomy	8186	55.9	1139	31.9	4178	63.1	2869	64.5
Wide local excision	5127	35.0	2423	67.9	1639	24.7	1065	23.9
Type of surgery unknown	1332	9.1	9	0.3	809	12.2	514	11.6
*Radiation therapy and chemotherapy* ^b^								
None	1663	11.4	439	12.3	578	8.7	646	14.5
Radiation therapy alone	8137	55.6	2513	70.4	3502	52.9	2122	47.7
Chemotherapy alone	406	2.8	19	0.5	216	3.3	171	3.8
Radiation therapy and chemotherapy	4439	30.3	600	16.8	2330	35.2	1509	33.9
*Radiation fields* ^b^								
No radiation therapy	2069	14.2	458	12.8	794	12.0	817	18.4
Breast, no IMC	6301	43.0	621	17.4	3285	49.6	2395	53.8
Typical mean heart dose left/right (Gy)	4.8/0.6 Gy		4.3/0.6 Gy		4.8/0.7 Gy		1.5/0.3 Gy	
Chest wall, no IMC	796	5.4	337	9.4	382	5.8	77	1.7
Typical mean heart dose left/right (Gy)	5.8/2.8 Gy		4.0/2.8 Gy		6.3/2.8 Gy		1.5/0.3	
IMC, no chest wall or breast	2269	15.5	1164	32.6	850	12.8	255	5.7
Typical mean heart dose left/right (Gy)	14.7/8.9 Gy		12.2/8.9 Gy		16.5/9.9 Gy		16.1/9.4 Gy	
IMC and breast	1429	9.8	475	13.3	679	10.3	275	6.2
Typical mean heart dose left/right (Gy)	16.6/13.4 Gy		16.6/15.3 Gy		21.8/13.4 Gy		9.1/9.2 Gy	
IMC and chest wall	806	5.5	430	12.0	226	3.4	150	3.4
Typical mean heart dose left/right (Gy)	16.1/10.1 Gy		14.8/12.6 Gy		16.4/10.5 Gy		16.1/1.7 Gy	
Unknown	975	6.7	86	2.4	410	6.2	479	10.8
*Chemotherapy regimen* ^b^								
No	9800	66.9	2952	82.7	4080	61.6	2768	62.2
CMF-like regimens	2029	13.9	619	17.4	1422	21.5	0	0
Anthracycline-based regimens^c^	2816	19.2	0	0	1124	17.0	1680	37.8
*Endocrine therapy* ^b^								
No	12,205	83.3	3503	98.1	6.043	91.2	2659	59.8
Yes	2440	16.7	68	1.9	583	8.8	1789	40.2
*Cardiovascular risk factors at breast cancer diagnosis* ^d^								
None known	10,908	74.5	1875	52.5	5132	77.5	3901	87.7
Hypertension, hypercholesterolemia or diabetes mellitus	671	4.6	355	9.9	186	2.8	130	2.9
Smoking^e^	2966	20.3	1265	35.4	1326	20.0	375	8.4
History of cardiovascular disease	484	3.3	315	8.8	82	1.3	97	2.0
*Follow-up time (years)*								
Median (IQR)	14 (9–20)		23 (17–28)		15 (9–19)		9 (6–11)	
1–4 years	1297	9.8	0	0	917	15.1	380	10.6
5–9 years	2604	19.7	0	0	723	11.9	1881	52.6
10–19 years	5816	44.0	1344	37.7	3154	52.0	1318	36.8
20–29 years	2979	22.5	1702	47.7	1277	21.1	0	0
≥30 years	523	4.0	523	14.7	0	0	0	0
*Vital status*								
Alive	10,064	68.7	1889	52.9	4240	64.0	3935	88.5
Deceased	4580	31.3	1682	47.1	2385	36.0	513	11.5

*IQR* interquartile range, *IMC* internal mammary chain.

^a^Median age for patients aged <35 years at diagnosis was 32 years, with an interquartile range of 30–34 years.

^b^Mutually exclusive treatment groups, taking into account primary treatment only.

^c^Including either epirubicin or doxorubicin.

^d^335 patients had more than one of the mentioned cardiovascular risk factors at breast cancer diagnosis and these patients are listed more than once. The most frequent combinations involved current or previous smoking.

^e^Smoking defined as quit shortly before breast cancer diagnosis, smoker at breast cancer diagnosis or smoker during follow-up. 17.5 % of the cohort had never smoked. Smoking information was missing for 62.3% of the cohort

### General population comparisons

Compared to the general population, our cohort had a higher MI rate (SIR = 1.4 95% CI 1.3–1.6), whereas the HF rate was not increased overall (SIR = 1.0 95% CI 0.9–1.1) (Table 2). While for HF the highest SIRs were seen for young ages at BC diagnosis, MI rates were increased only for older ages at diagnosis (Table 2). Subdividing the entire cohort by follow-up duration and treatment period, an increased HF rate was observed 1–9 years after treatment in patients treated ≥1987 (SIR = 1.4 95% CI 1.1–1.9 for 1987–1999 and 1.5 95% CI 1.0–2.0 for 2000–2009). In contrast, the increases in the MI rate were greatest in the longest follow-up intervals.

**Table 2:** Comparison of myocardial infarction and heart failure rates with the general population

	Myocardial infarction^a^				Heart failure^a^			
	Observed	SIR	95% CI	AER	Observed	SIR	95% CI	AER
Total	394	1.4	1.3–1.6	8	396	1.0	0.9–1.1	0
*Age at breast cancer diagnosis (years)*								
<35	5	0.9	0.3–2.1	0	12	2.7	1.4–4.7	7
35–40	17	1.1	0.7–1.8	1	20	1.4	0.9–2.2	4
40–49	180	1.5	1.3–1.7	8	179	1.1	1.0–1.3	3
50–61	192	1.4	1.2–1.6	12	185	0.8	0.7–1.0	−8
*Calendar period of breast cancer diagnosis and follow-up interval*								
1970–1986								
10–19 years	128	1.3	1.1–1.5	21	91	0.8	0.7–1.0	−16
20+ years	120	2.1	1.7–2.5	210	127	0.9	0.7–1.0	−63
1987–1999								
1–9 years	41	0.7	0.5–1.0	-6	57	1.4	1.1–1.9	8
10–19 years	54	1.7	1.3–2.2	15	64	1.1	0.8–1.4	3
20+ years	8	1.7	0.7–3.4	24	9	0.8	0.4–1.5	−17
2000–2009								
1–9 years	26	1.5	1.0–2.2	7	36	1.5	1.0–2.0	9
10+ years	6	2.0	0.7–4.3	23	12	2.6	1.3–4.5	58
*Radiation therapy and chemotherapy*								
None	29	0.8	0.5–1.1	−5	33	0.5	0.4–0.8	−16
Radiation therapy alone	264	1.5	1.4–1.7	12	233	0.9	0.7–1.0	−5
Chemotherapy alone	6	2.6	0.9–5.5	13	8	2.7	1.2–5.3	16
Radiation therapy and chemotherapy	75	1.7	1.4–2.2	9	122	2.1	1.7–2.5	16
*Radiation fields* ^b^								
Breast (no IMC)	87	1.2	0.9–1.4	2	81	0.8	0.6–1.0	−3
Chest wall (no IMC)	34	1.5	1.0–2.0	14	42	1.0	0.7–1.3	−1
IMC	203	1.9	1.6–2.1	23	205	1.2	1.0–1.4	6
*Chemotherapy regimens*								
CMF-like regimens	59	1.7	1.3–2.2	11	44	1.0	0.8–1.4	0
Anthracycline-based regimens^c^	22	1.5	0.9–2.2	3	86	4.6	3.7–5.7	33
*Cardiovascular risk factor at BC diagnosis* ^d^								
None known	342	1.3	1.2–1.5	6	347	1.0	0.9–1.1	−1
At least one	52	2.3	1.7–3.0	42	49	1.3	1.0–1.8	17
*Smoking*								
Never	110	1.1	0.9–1.3	3	115	0.8	0.6–0.9	−10
Currently or previous	174	2.3	2.0–2.7	28	141	1.4	1.2–1.6	11
Unknown	110	1.0	0.8–1.2	0	140	0.9	0.8–1.1	−1

*SIR* standardised incidence ratio, *CI* confidence interval, *AER* absolute excess risk, *IMC* internal mammary chain.

^a^Expected numbers were calculated using age-, sex- and calendar period-specific CVD incidence rates for the Dutch population. Myocardial infarction and heart failure incidence data from the Continuous Morbidity Registration Nijmegen of General Practices were used as reference rates for the years 1971–1999 and from the Netherlands Institute for Health Services Research Primary Care Database from 2000 onwards. Myocardial infarction included diagnoses I21–22 International Classification of Diseases, 10th revision. Heart failure included both cardiomyopathy and congestive heart failure; diagnoses I42 and I50 International Classification of Diseases, 10th revision. These were the only two cardiovascular diseases for which general population data were available. Just as in the general population registries, each individual patient in our cohort could have had a diagnosis of both myocardial infarction and heart failure.

^b^Mutually exclusive treatment categories.

^c^Including either epirubicin or doxorubicin.

^d^Hypertension, hypercholesterolaemia or diabetes mellitus

Among patients treated with neither RT nor chemotherapy, the MI rate was not increased (SIR = 0.8 95% CI 0.5–1.1) and the HF rate was decreased (SIR = 0.5 95% CI 0.4–0.8) compared with the general population. Increased MI rates were observed after RT (e.g. SIR = 1.5 95% CI 1.4–1.7 for patients treated with RT and without chemotherapy), while HF rates were increased after anthracycline-based chemotherapy (SIR = 4.6 95% CI 3.7–5.7).

### Within-cohort comparisons

For women treated with RT, the lowest typical mean heart doses were for those who received right-sided breast irradiation without IMC (0.6 Gy, interquartile range (IQR) 0.3–0.7) (Table 1, Supplementary table 1). Compared to this group, women who received IMC irradiation (either left- or right-sided, average of mean heart doses for typical IMC irradiation 12.2 Gy, IQR 8.7–16.5) had significantly increased rates of all four cardiovascular outcomes: any CVD (hazard ratio (HR) = 1.56 95% CI 1.35–1.84), IHD (HR = 2.36 95% CI 1.74–3.22), VHD (HR = 1.63 95% CI 1.18–2.24) and HF (HR = 1.82 95% CI 1.27–2.63, based on inclusion of multiple CVDs per woman) (Summary model, Table 3). Increases were observed after both left- and right-sided IMC (Table 3) and with/without additional breast or chest wall radiation (Supplementary table 2). Increased rates of any CVD and of IHD were also seen after left chest wall irradiation (average of typical mean heart doses 5.8 Gy, IQR 3.8–5.3) when compared to right breast irradiation (HRs were 1.83 95% CI 1.39–2.40 and 2.57 95% CI 1.61–4.11, respectively). In the entire cohort, no significant increases were observed in women with left breast irradiation (average of mean heart doses 4.7 Gy, IQR 1.5–4.8) compared to those treated with right breast irradiation (HR for IHD 1.38 95% CI 0.96–1.99, Supplementary table 2); yet, for women treated at age ≤50 years an increased rate of IHD was observed (HR = 1.70 95% CI 1.03–2.80) (Supplementary table 3). Additional analyses considered just the first cardiovascular event and found the following (very similar) HRs for women who received IMC irradiation compared with women who received right-sided breast irradiation without IMC: any CVD (HR = 1.49 95% CI 1.25–1.77), IHD (HR = 2.51 95% CI 1.70–3.72), VHD (HR = 1.57 95% CI 1.02–2.44) and HF (HR = 1.71 95% CI 0.99–2.94) (Supplementary table 4).

**Table 3:** Within-cohort comparison of cardiovascular disease rates after breast cancer by treatment

	Any cardiovascular event			Ischaemic heart disease ≥10 years after breast cancer treatment^a^			Valvular heart disease			Heart failure^b^		
	*n*/*N*^*d*^	HR	(95% CI)	*n*/*N*^d^	HR	(95% CI)	*n*/*N*^d^	HR	(95% CI)	*n*/*N*^d^	HR	(95% CI)
**Multivariable model** ^c^												
*Radiation field* ^f^												
Breast, right-sided (no IMC)	230/2562	1.00	Ref.	48/1684	1.00	Ref.	51/2519	1.00	Ref.	40/2520	1.00	Ref.
Chest wall, right-sided (no IMC^e^)	61/315	1.24	0.93–1.65	24/244	1.73	1.05–2.85	10/349	0.51	0.25–1.03	23/350	1.68	0.98–2.88
IMC, right-sided (+/− breast/chest wall)	344/1804	1.50	1.26–1.78	180/1478	2.54	1.84–3.52	97/1824	1.26	0.88–1.79	90/1824	1.78	1.21–2.61
Breast, left-sided (no IMC)	272/2761	1.11	0.93–1.32	70/1821	1.37	0.95–1.98	56/2797	1.00	0.69–1.47	41/2798	0.87	0.56–1.35
Chest wall, left-sided (no IMC^e^)	71/302	1.83	1.39–2.40	96/226	2.57	1.61–4.11	16/352	0.91	0.50–1.62	20/352	1.42	0.80–2.50
IMC, left-sided (+/− breast/chest wall)	413/1963	1.66	1.41–1.97	190/1621	2.20	1.59–3.04	162/2002	2.00	1.44–2.78	118/2002	1.94	1.33–2.82
No radiation therapy	221/1825	1.21	1.00–1.46	72/1222	1.50	1.04–2.17	44/1738	0.78	0.52–1.18	44/1741	1.22	0.79–1.89
*Chemotherapy* ^f^												
No chemotherapy	1258/8238	1.00	Ref.	506/6112	1.00	Ref.	336/8296	1.00	Ref.	274/8301	1.00	Ref.
CMF-like regimen	240/1727	1.00	0.87–1.16	105/1363	1.07	0.85–1.33	72/1751	1.15	0.88–1.50	44/1749	0.89	0.64–1.24
Anthracycline-based regimen	193/2252	1.51	1.25–1.82	21/1107	1.00	0.61–1.64	43/2262	1.75	1.16–2.65	84/2263	4.32	3.07–6.07
*Endocrine therapy*												
No endocrine therapy	1518/10201	1.00	Ref.	605/7614	1.00	Ref.	406/10283	1.00	Ref.	345/10286	1.00	Ref.
Endocrine therapy	173/2016	0.97	0.80–1.17	27/968	0.85	0.55–1.29	45/2026	1.22	0.83–1.79	57/2027	0.93	0.65–1.31
**Summary model** ^h^												
Breast, right-sided (no IMC)	230/2562	1.00	Ref.	48/1684	1.00	Ref.	51/2519	1.00	Ref.	40/2520	1.00	Ref.
IMC (left- or right-sided, +/− breast/chest wall)	757/3629	1.56	1.35–1.84	370/3099	2.36	1.74–3.22	259/3826	1.63	1.18–2.24	208/3826	1.82	1.27–2.63
**Joint effects of treatments** ^f,g^												
Breast RT (no IMC), no anthracyclines	441/4475	1.00	Ref.	111/3102	1.00	Ref.	97/4423	1.00	Ref.	59/4312	1.00	Ref.
IMC RT, no anthracyclines	690/3113	1.54	1.35–1.75	361/2697	2.00	1.50–2.66	242/3159	1.74	1.35–2.25	165/2993	2.14	1.55–2.96
Breast RT (no IMC), anthracyclines	61/848	1.52	1.16–1.99	7/402	1.88	0.90–3.93	10/893	1.24	0.64–2.40	20/941	5.10	3.12–8.34
IMC RT, anthracyclines	67/654	2.09	1.62–2.69	9/402	2.32	1.19–4.55	17/667	2.86	1.76–4.65	31/683	9.23	6.01–14.18
Test for departure from additivity/multiplicativity		*p* = 0.70/0.74			*p* = 0.57/0.27			*p* = 0.51/0.96			*p* = 0.06/0.68	

*n/N* number of events/number at risk, *HR* hazard ratio, *CI* confidence interval, *IMC* internal mammary chain, *Ref.* reference category.

The analyses shown in this table include all diagnoses of cardiovascular disease, e.g. if a patient was diagnosed with ischaemic heart disease and then later with valvular heart disease, then both are listed. Analyses considering just the first diagnosis of cardiovascular disease are in Supplementary Table 4.

^a^Because the proportional hazard assumption did not hold for the ischaemic heart disease rate after internal mammary chain and chest wall irradiation, results are shown here for ≥10 years after breast cancer treatment. No increased ischaemic heart disease rates were seen in the period <10 years after treatment. These results are presented in Supplementary Table 5.

^b^Heart failure included both cardiomyopathy and congestive heart failure; diagnoses I42 and I50 International Classification of Diseases, 10th revision.

^c^Hazard ratios estimated using one multivariable model containing radiation fields (right breast, right-sided chest wall, right-sided internal mammary chain field, left breast, left-sided chest wall, left-sided internal mammary chain field, no radiation therapy, unknown radiation fields), chemotherapy (no chemotherapy, CMF-like regimen, anthracycline-based regimen), endocrine therapy (no, yes), age at breast cancer treatment (<40, 40–49, 50–61 years), cardiovascular risk factor at breast cancer diagnosis yes/no (hypertension, hypercholesterolemia or diabetes), smoking (ever, never or unknown) and other cardiovascular diseases (time-dependent). Hazard ratios for the covariates, estimates for patients with unknown radiation fields and estimates for patients irradiated to the internal mammary chain separately for patients additionally irradiated to the breast/chest wall are shown in Supplementary Table 2.

^d^Analyses included all patients with at least 1 day of cardiovascular follow-up after start of time at risk (*n* = 12,355). Patients with a specific cardiovascular diagnosis before start of time at risk were excluded from analysis with that specific diagnosis as end point (*n* = 138 for any cardiovascular event [including also 27 diagnoses of arrhythmia and 3 of pericarditis], *n* = 50 for ischaemic heart disease, *n* = 18 for valvular heart disease and *n* = 36 for heart failure). Numbers at risk differs by end point due to time-dependency of the treatment variables.

^e^For some women who were treated with direct electrons with the chest wall as the target, the internal mammary chain received a therapeutic dose.

^f^Mutually exclusive treatment categories, taking into account primary treatment, as well as treatment for (loco)regional recurrences and second breast cancers.

^g^Hazard ratios estimated using one multivariable model containing one variable for the joint effect of radiation therapy and anthracycline-based chemotherapy (breast irradiation without anthracycline-based chemotherapy, internal mammary chain irradiation without anthracycline-based chemotherapy, breast irradiation with anthracycline-based chemotherapy, internal mammary chain irradiation with anthracycline-based chemotherapy), age at breast cancer (<40, 40–50, 50–61 years), cardiovascular risk factor at breast cancer diagnosis yes/no (hypertension, hypercholesterolaemia or diabetes), smoking (ever, never or unknown) and other cardiovascular diseases (time-dependent). Patients not irradiated to either the breast or internal mammary chain were excluded from these analyses.

^h^Hazard ratios estimated using one multivariable model containing radiation fields (right breast, right-sided chest wall, left breast, left-sided chest wall, internal mammary chain [left- or right-sided], no radiation therapy, unknown radiation fields), chemotherapy (no chemotherapy, CMF-like regimen, anthracycline-based regimen), endocrine therapy (no, yes), age at breast cancer treatment (<40, 40–49, 50–61 years), cardiovascular risk factor at breast cancer diagnosis yes/no (hypertension, hypercholesterolemia or diabetes), smoking (ever, never or unknown) and other cardiovascular diseases (time-dependent)

Women treated with anthracycline-based chemotherapy had increased rates of VHD (HR = 1.75 95% CI 1.16–2.65) and HF (HR = 4.32 95% CI 3.07–6.07) compared to no chemotherapy (Table 3, based on inclusion of multiple CVDs per woman). When just the first cardiovascular diagnosis was considered, the increase in HF was slightly reduced (HR = 3.93 95% CI 2.49–6.22) (Supplementary table 4). When including VHD events diagnosed on the same day as IHD/HF, the anthracycline-based chemotherapy-associated risk of VHD was still increased (HR = 1.70 95% CI 1.09–2.65), but when excluding such VHD events the HR dropped to 1.11 (95% CI 0.62–2.00). Additional stratification by treatment period did not affect the estimates (results not shown). No increased CVD rates were observed comparing patients treated with endocrine therapy to no endocrine therapy.

The joint effects of IMC irradiation, anthracycline-based chemotherapy, cardiovascular risk factors at BC diagnosis and smoking were compatible with either an additive or a multiplicative relation for all CVDs (Supplementary table 6). For HF, however, the combined effect of IMC irradiation and anthracycline-based chemotherapy seemed more than additive (*p* = 0.06). A more than nine-fold increase was observed among patients treated with both IMC irradiation and anthracycline-based chemotherapy (HR = 9.23 95% CI 6.01–14.18), whereas the separate HRs were 2.14 (95% CI 1.55–2.96) and 5.10 (95% CI 3.12–8.34), respectively, all compared to either IMC or anthracycline-based chemotherapy (Table 3).

**Fig. 1:**
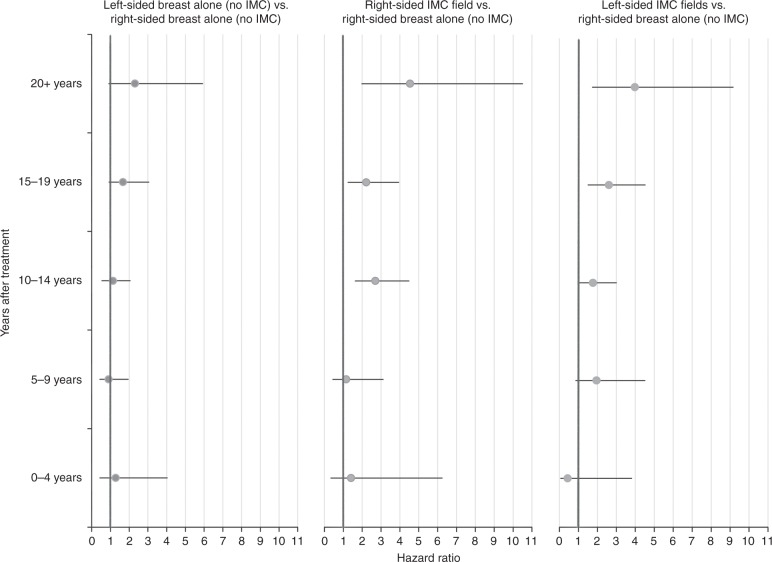
Within cohort comparison of ischemic heart disease rates by time since treatment and radiation therapy in patients diagnosed during 1970-1999. The analyses shown in this figure include all diagnoses of ischemic heart disease, e.g. including patients diagnosed with valvular heart disease or heart failure prior to ischemic heart disease. For women diagnosed with breast cancer during 1970-86, data on cardiovascular disease were available only for the period 10+years after treatment. IMC, internal mammary chain.Cox proportional hazard model including the following variables: radiation fields (right-), age at breast cancer treatment (<40, 40-49, 50-61 years), chemotherapy (none, CMF-like, anthracycline-based chemotherapy), cardiovascular risk factor at breast cancer diagnosis yes/no (hypertension, hypercholesterolemia, or diabetes), smoking (ever, never, or unknown), and other cardiovascular diseases diagnoses (time-dependent).In the period 2000-2009 follow-up duration was too short for reliable estimates (see Table 4)

When analysing IHD rates by time since treatment, no significant increases were seen in the first 10 years (Fig. 1, Supplementary table 5). IMC irradiation during 1970–1986 or 1987–1999 was associated with increased IHD rates ≥10 years after treatment (Fig. 1, Table 4); the HR for 0–9 years after IMC irradiation compared to breast irradiation only during 1987–1999 was 1.32 (95% CI 0.74–2.37), while for 10+ years the HRs were 1.64 (95% CI 1.19–2.25) and 1.72 (95% CI 1.17–2.53) for 1970–1986 and 1987–1999, respectively. In the period 2000–2009, numbers were too small to detect or reject a risk increase for either 0–9 or ≥10 years after IMC irradiation (Table 4). HF rates after anthracycline-based chemotherapy were increased compared to no chemotherapy during the period 1–4 years after diagnosis (HR = 6.80 95% CI 2.75–16.82) and remained increased until at least 10–15 years after treatment (HR = 4.03 95% CI 2.70–6.00).

**Table 4:** Within-cohort comparison of ischaemic heart disease ratios for different radiation fields by time since treatment and treatment period

Treatment period		Time since treatment								
Radiation field^a^		0–9 years			10–19 years			20+ years		
		*n*/*N*	HR	(95% CI)	*n*/*N*	HR	(95% CI)	*n*/*N*	HR	(95% CI)
*1970–1986*										
Breast only (no IMC)		0/0	—		44/1162	1.00	Ref.	11/402	1.00	Ref.
IMC^b^		0/0	—		318/3899	1.35	0.93–1.96	144/1455	2.51	1.35–4.67
*1987–1999*										
Breast only (no IMC)	37/3345		1.00	Ref.	66/3432	1.00	Ref.	10/784	1.00	Ref.
IMC^b^	20/1524		1.32	0.74–2.37	48/1340	1.68	1.09–2.57	9/261	2.11	0.85–5.25
2000-2009										
Breast only (no IMC)	34/2,028		1.00	Ref.	8/686	1.00	Ref.	0/0	—	
IMC^b^	5/544		0.62	0.23–1.62	4/290	0.90	0.26–3.05	0/0	—	

*n/N* number of events/number at risk, *HR* hazard ratio, *CI* confidence interval, *IMC* internal mammary chain, *Ref.* reference category.

^a^Patients were time-dependently categorised based on the treatment they received throughout follow-up into irradiation of the breast without internal mammary chain irradiation (either left or right breast), internal mammary chain irradiation (left- of right-sided) with or without radiation of additional fields and no/other radiation fields (estimates not shown).

^b^Irradiation of the left- or right-sided internal mammary chain, with or without additional irradiation of the breast or chest wall

**Fig. 2:**
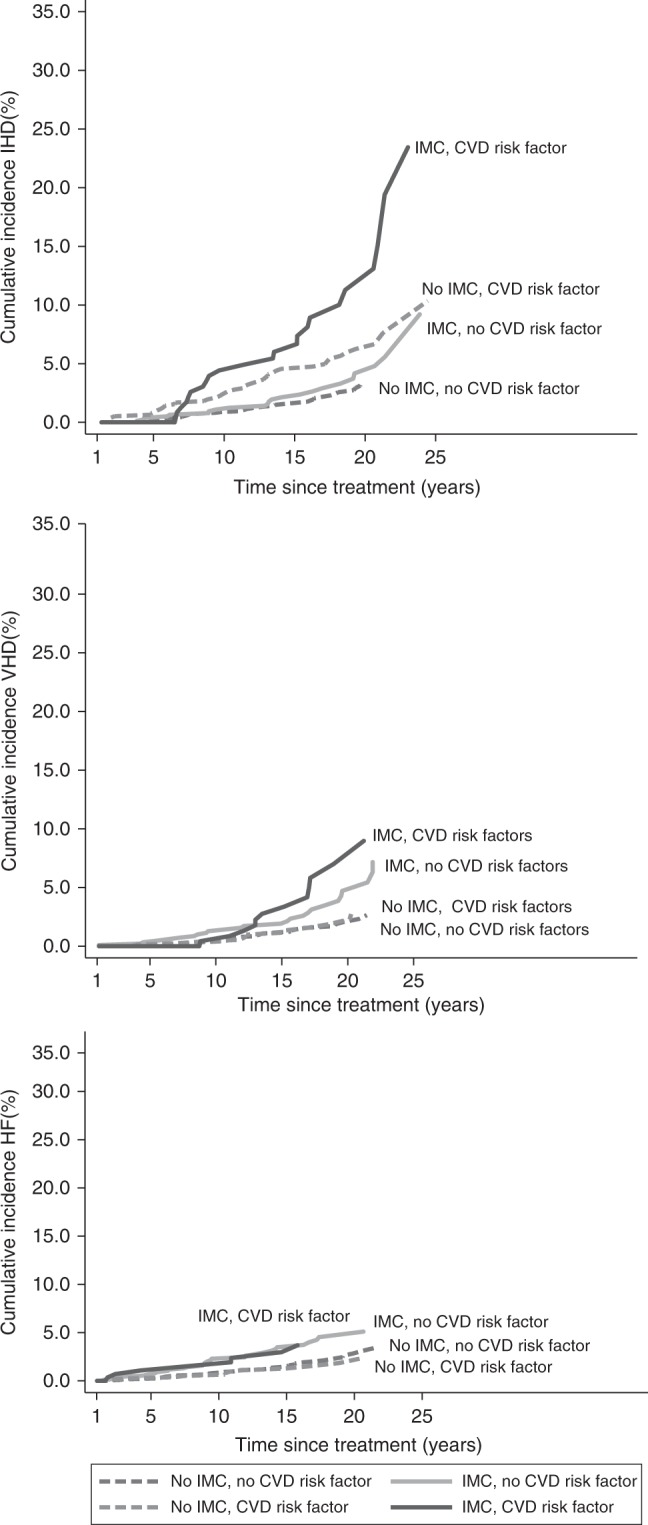
Cumulative risk of cardiovascular diseases in patients diagnosed during 1987-1999 and aged 50 years or younger at breast cancer diagnosis, by internal mammary chain irradiation and cardiovascular disease risk factors (including smoking) at breast cancer diagnosis. IMC, internal mammary chain; CVD, cardiovascular disease; CHD, ischemic heart disease; VHD, valvular heart disease; HF, heart failure. The analyses of ischemic heart disease, valvular heart disease, and heart failure shown in this figure include all diagnoses of cardiovascular disease, e.g. if a patient was diagnosed with ischemic heart disease and then later with valvular heart disease then both events are counted. Patients with a specific cardiovascular diagnosis before start of time at risk were excluded from analysis with that specific diagnosis as endpoint (n=50 for ischemic heart disease, n=18 for valvular heart disease, and n=36 for heart failure)

Among women diagnosed before age 50 years during 1987–1999, the cumulative incidence of IHD twenty years after BC treatment was 11.3% (95% CI 6.8–17.1) for those who received IMC irradiation and had a cardiovascular risk factor (including smoking) at diagnosis compared to 6.4% (95% CI 4.5–8.7) for those who had a cardiovascular risk factor but did not receive IMC irradiation (Fig. 2). For VHD and HF, the cumulative 20-year incidences were also considerably higher for women who received IMC radiation and had a cardiovascular risk factor compared with those who had a cardiovascular risk factor but no IMC radiation. Results for age 50+ years are in Supplementary Figure 1. Cumulative incidences of IHD, VHD and HF by anthracycline-based treatment and IMC irradiation for women aged ≤50 years are given in Supplementary Figure 2.

Women treated more recently (1990–2006) had a lower cumulative MI risk than women treated in earlier years (1970–1989) (Supplementary Figure 3). In addition, the absolute increase in cumulative MI risk compared to the population-expected risk was notably smaller for those treated during 1990–2006 than for those treated before 1990. When compared with women receiving right breast RT only, the HR for all other RT regimens was 2.82-fold (95% CI 1.48–5.37) for the period 1980–1989 and 1.84 (95% CI 0.83–4.05) for the period 1990–2006 (10-year survivors only; *p*_difference_ = 0.41).

## Discussion

Our study shows that, in women treated for BC in the Netherlands between 1970 and 2009, IMC irradiation was associated with an increased incidence of IHD, VHD and HF. Risk increases were seen not only after left-sided but also after right-sided IMC irradiation, and importantly, the proportional increase in the risk of IHD was greatest in the period >20 years after treatment. Anthracycline-based chemotherapy was associated with increased incidence of HF. The combination of IMC irradiation and anthracycline-based chemotherapy was associated with a nine-fold increased incidence of HF relative to patients who received only breast RT and no anthracycline-based chemotherapy.

Anthracycline-based chemotherapy (received by women in our cohort after 1990) was associated with increased HF incidence up to 15 years after treatment; there was insufficient follow-up to assess risk beyond this. Our estimate of the proportional increase in the rate (HR = 4.32) is somewhat higher than previously reported in population-based studies.^21,39,40^ A possible explanation is the young age of the women in our cohort, as we observed an even larger increases in patients treated ≤50 years (HR = 5.23 95% CI 3.41–8.01).

The increased VHD rate after anthracycline-based chemotherapy when multiple CVD diagnoses per woman are considered is a new finding in BC patients. Our detailed analysis, however, excluding VHD events diagnosed at the time of HF/IHD diagnosis suggests that the anthracycline-based chemotherapy-associated VHD risk in this cohort may be caused by anthracycline-based chemotherapy-related HF, rather than a direct effect of anthracycline-based chemotherapy. An anthracycline-related increase in the diagnosis of VHD as a first CVD event has previously been observed in Hodgkin lymphoma patients.^41,42^

In a recent case–control study, the risk of a major coronary event increased by 7.4%/Gy mean heart dose.^8^ Although not statistically significant, our HR of 1.38 for left breast (~5 Gy typical mean heart dose) versus right breast RT (~0.6 Gy typical mean heart dose) is consistent with these results. In our large, population-based cohort of early BC patients,^43^ we studied hospitalisation for CVD and also found an increased rate of IHD comparing left- versus right-sided breast irradiation (without IMC irradiation) (HR = 1.24 95% CI 1.01–1.52). These findings, together with the increased rate we observed in patients treated at age ≤50 years in the current study, suggest that left breast irradiation does slightly increase IHD risk. Also in line with Darby and colleagues’ results are our IHD HRs of 1.77–2.78 for women who received typical heart doses of ~9–15 Gy from IMC RT compared with women with right breast RT. The effect of cardiovascular risk factors on radiation-related cardiac risk in the two studies is also consistent. In both studies, cardiovascular risk factors prior to RT did not significantly increase *relative* risk of radiation-related CVD but did increase the *absolute* risk due to RT. Our study included patients up to the age of 61 years at BC diagnosis. Older patients generally have more cardiovascular risk factors. Hence, the absolute risks of treatment-related CVD may be higher in older patients. Additionally, the presence of cardiovascular risk factors might influence the onset of treatment-related CVD. Future studies should focus also on older BC patients and the onset of the increased CVD rates among these older patients.

Our results are relevant to a large number of BC survivors treated with older IMC regimens, who may remain at an elevated CVD risk for an extensive period. Follow-up in our study was too short to detect or reject an IHD risk increase associated with IMC irradiation during 2000–2009. Recent studies showing improved BC survival after IMC irradiation^24,25,44^ still have insufficient follow-up (≤10 years) to detect an increased CVD risk which, as we report, continues into the third decade after treatment. For women who receive BC treatment today, the predicted absolute risks of IMC RT are expected to be substantially lower than for the women in our study. This is partly because the IHD risk in the general population has decreased substantially since the 1970s (Supplemental Figure 3). A recent systematic review of heart dose estimates from BC RT during 2003–2013 showed that heart dose from IMC regimens varied according to technique and was typically ~8 Gy in left-sided RT^27^, which is lower than the average of ~13 Gy in our study. Modern radiotherapy techniques, including intensity-modulated radiotherapy and deep-inspirational breath hold, can deliver mean heart doses of <4 Gy even for IMC radiotherapy in left-sided tumours and their use is strongly recommended.

Our results suggest that the combined effects of radiation and anthracycline-based chemotherapy may be greater than their individual effects on the heart. This finding needs confirmation as in several countries guidelines recommend both IMC RT and anthracycline-based chemotherapy sequentially for women with poor prognostic features, such as nodal involvement.

Strengths of our study include data on RT fields and type of chemotherapy, GP- and cardiologist-reported CVD incidence and cardiovascular risk factors and long and near-complete follow-up. Surveillance bias in our study population is unlikely, as there are no recommendations concerning CVD screening in the nation-wide to BC follow-up guidelines in the Netherlands, which are adhered to closely.

A potential limitation that we have considered is whether the increased CVD risk associated with BC treatment might be due to a less favourable cardiovascular risk profile among women who received IMC radiation or anthracycline-based chemotherapy and this in turn might be associated with higher BC stage and lower socioeconomic status. However, in our relatively young BC cohort from two cancer centres, BC treatment was not associated with socioeconomic status, cardiovascular history at BC diagnosis or cardiovascular risk factors. Data on other risk factors for CVD, such as family history of CVD, body mass index and chronic obstructive pulmonary disease, were, unfortunately, not collected. However, in the Netherlands, BC treatment guidelines do not recommend taking CVD risk factors into account and, accordingly, no differences in prevalence were observed between the treatment categories for the CVD risk factors that were collected. Therefore, missing information on other CVD risk factors is unlikely to have affected our estimates. Another potential limitation is the possibility of unreported events. Because, inherent to a retrospective study design, we rely on the registration of events in medical records, it is possible that some CVD events might have gone unreported. This might have caused our estimates to be slightly underestimated. Lastly, our study did not include patients treated with trastuzumab or taxanes, nor were we able to consider the different types of endocrine therapy. CVD rates after these modern systemic therapies should be evaluated in future studies.

In conclusion, anthracycline-based chemotherapy and irradiation using regimens with substantial mean heart doses (9–17 Gy) were associated with increased incidence of several types of CVDs. The predicted absolute risks of IMC RT are lower for women today, and for most women, the benefits will exceed the risks. However, the risks may be greater for some subgroups, e.g. women with left-sided BC who receive both IMC irradiation and anthracycline-based chemotherapy or who have cardiovascular risk factors. For BC survivors, our results are also relevant as subgroups may benefit from cardiac surveillance.^45^

## Electronic supplementary material

Supplementary material
