# Supplementary material for: Cardiovascular disease incidence after internal mammary chain irradiation and anthracycline-based chemotherapy for breast cancer
Source: Br J Cancer. 2018 Aug 1;119(4):408–18. doi: 10.1038/s41416-018-0159-x (PMC6133926; doi:10.1038/s41416-018-0159-x)
Supplement: Supplementary file 1 — Supplementary material [file 41416_2018_159_MOESM1_ESM.docx]

**Supplementary material - ONLINE ONLY**

**Supplementary Methods I: Patient selection procedures**

Female breast cancer patients (stage I-IIIA or ductal carcinoma in situ [DCIS]) were selected from the hospital-based registries of the Netherlands Cancer Institute, Amsterdam or the Erasmus MC - Cancer Institute, Rotterdam, the Netherlands. All patients had to have received at least surgery. Patients who had previously been treated with radiation therapy between the diaphragm and the chin, or treated with any type of chemotherapy before breast cancer diagnosis were not included in the cohort. Data collection from the registries and medical files included the following variables: date of birth, breast cancer diagnosis, tumour histology, stage, type of surgery, radiation fields, chemotherapy regimen, hormonal treatment, date of first recurrence and distant metastasis, date, diagnosis and treatment of previous and subsequent malignancies, history of CVD before breast cancer diagnosis, dates and diagnoses of cardiovascular events, smoking, hypertension, diabetes mellitus, hypercholesterolemia, date of last known medical status, and cause of death (according to the International Classification for Diseases, 10th revision).

Because data collection on cardiovascular disease incidence through questionnaires to general practitioners and cardiologists is labour intensive, and because we were interested in long-term cardiovascular disease risks following radiation therapy and chemotherapy, the current study was restricted to patients who were diagnosed with breast cancer before the age of 62 years (in the study period very few patients older than 62 years received chemotherapy). When the first part of the cohort was established in the 1990s, it was generally assumed that increased cardiovascular risks did not emerge until the second decade after breast cancer treatment. Hence, for patients diagnosed between 1970 and 1986, cardiovascular disease information was acquired only for ten-year survivors[^1^](#_ENREF_1). In addition, because during this period the majority of patients were treated with surgery plus radiation therapy, for reasons of efficiency, a stratified sample was taken of all patients treated with surgery plus radiation therapy, stratified by age. For all other treatment combinations, all ten-year survivors were included in the study. When the cohort was expanded with patients diagnosed between 1987 and 2009, we were also interested in the possible risk of cardiovascular disease after anthracyclines, which were thought to occur earlier after breast cancer treatment than radiation therapy effects. For patients diagnosed between 1987 and 2009, we therefore aimed to collect cardiovascular disease information for all one-year survivors. However, because funding resources were limited, we were obliged to make a patient selection for part of the expansion; the years of diagnosis 1994 to 2000. For patients diagnosed during this period we again took a stratified sample of all patients treated with surgery plus radiation therapy, stratified by age. For other treatment combinations, all one-year survivors were included. For the years of diagnosis 1987 to 1993 and 2001 to 2009 all one-year survivors were included. Selection of patients was always random within each age stratum and by definition independent of cardiovascular disease diagnosis, as the hospital-based registries did not contain any data on cardiovascular disease.

To complete cardiovascular follow-up in the entire cohort, letters were sent to general practitioners and cardiologists. In The Netherlands, all residents are expected to have a general practitioner. Medical correspondence from attending physicians is sent to the general practitioner. Records are preserved by the general practitioner throughout a patient’s life and for at least 15 years after a patient’s death. Hence, questionnaires were sent to general practitioners of all patients who were alive at last follow-up and to all patients who had died less than 15 years previously. For patients treated before 2000, complete follow-up information to January 1, 2009 or later was available for 82% of the study cohort. For patients treated in 2000-2009, complete follow-up information to January 1, 2012 or later was available for 71% of the study cohort. For the other patients, the current/last general practitioner was unknown to us or unwilling to participate in the study. Selection bias introduced by general practitioners is unlikely as patient information did not appear to play a role in the decision to participate; for 61% of the patients with incomplete information, the GP was unknown or did not respond to any of the questionnaires. Less than 1% of patients were lost to follow-up because their medical files had been destroyed. Median follow-up duration was 14 years for the entire cohort; 23 years for patients diagnosed with breast cancer ≤1986 and 12 years for patients diagnosed >1986.

In the collection of established cardiovascular risk factors at BC diagnosis from the medical files, we had to rely on accurate reporting by oncologists. As very few patients had a history of cardiovascular disease at BC diagnosis, and the prevalence of cardiovascular risk factors at BC diagnosis did not differ by treatment (data not shown), random incompleteness is most likely. This may have attenuated the hazard ratios for women with cardiovascular risk factors relative to women without risk factors.

**References**

1. Hooning MJ, Botma A, Aleman BM, et al: Long-term risk of cardiovascular disease in 10-year survivors of breast cancer. J.Natl.Cancer Inst. 99:365-375, 2007

**Supplementary Methods II: Dosimetry**

Typical heart doses from the breast cancer radiation therapy regimens used in the Netherlands during the period that the women in the study cohort were treated were estimated by two of the authors (FD and CT). The estimates were based on dosimetry performed for a sample of 683 women. These were women (cases and controls) who had been selected for nested case-control studies of myocardial infarction and heart failure. Individual anatomical information was not available for them. CT-based radiotherapy planning was used only from 2005 onwards and so for <10% of the sample. Even where it was used, the CT-planning scans were not usually retrievable. Therefore the following method based on radiotherapy charts, which were available for all the women in the sample, was used for them all.

*Chart categorisation*

Information was abstracted from each woman's radiotherapy chart including: surgery type, target definition, field borders, total dose and dose per fraction, beam energy and the use of shielding, wedges and bolus. Each woman was then categorised according to the radiotherapy regimen she received. Fortyfour regimens were received by the 683 women, 22 regimens for left-sided and 22-regimens for right-sided breast cancer.

*Cardiac contouring*

Ten CT-planning scans were randomly selected from women referred for breast cancer radiotherapy in 2010. The treatment position for all women was supine, with both arms above the head. Slice thickness for each scan was 3 mm, and intravenous contrast was not used. The whole heart was contoured on each of the 10 scans. To simulate mastectomy the 10 CT planning scans were duplicated and the breast was virtually removed from the dose calculations.

*Selection of a 'typical CT-scan'*

The most commonly used left-sided regimen was identified from the charts as midline tangents using opposing symmetrical beams with a divergent posterior border. This regimen was reconstructed on each of the 10 CT scans and whole heart doses were reviewed. Anatomical features which may influence heart dose from breast cancer radiotherapy were measured including: sternal length, heart volume, chest wall separation distance and the Haller index (ratio of height between the anterior spine and posterior sternum to the transverse width of the heart). From these 10 CT scans, the selected 'typical CT-scan' was the scan with a mean heart dose closest to average which was not atypical for any of the anatomical factors reviewed: MHD “typical CT-scan”: 4.8 Gy, average MHD based on the 10 CT scans: 4.8 Gy (range 1.9 – 9.1 Gy).

*Regimen reconstruction*

All forty-four regimens were reconstructed on the 'typical CT-scan'. A 3-dimensional CT treatment planning system (Varian Eclipse^TM^ Treatment Planning System (TPS) version 10.0.39 (Varian Medical Systems, Palo Alto, USA)) was used to estimate heart doses from reconstructed cobalt, electron and megavoltage beams of varying energies. The analytical anisotropic, Monte Carlo and pencil beam algorithms were used to calculate dose for photon, electron and cobalt plans respectively. For each regimen a dose volume histogram for the whole heart was extracted. Manual planning was used to estimate heart doses from orthovoltage fields. Isodose charts were superimposed onto 10 axial CT images spanning the heart from top to bottom. A dose volume histogram for the whole heart was plotted for each orthovoltage regimen.

*Allocation of doses by laterality and irradiation of the internal mammary chain*

Each of the 683 women was allocated a typical mean heart dose based on her regimen and total dose. Women were then categorised according to laterality and whether they received IMC irradiation. Within these categories the typical doses were averaged.

*Limitations*

The true mean heart doses received by the individual women in the cohort undoubtedly differ substantially from the typical mean heart doses that have been estimated using the sample of 683 women by means of the method described above. In addition to sampling error, sources of variation include variation in patient anatomy, set-up error, inter- and intra-fraction motion, and delineation variation. The purpose of the estimates is solely to provide an indication of the potential level of cardiac exposure for women with left-sided and right-sided breast cancer who received radiotherapy to different targets during different time periods.

|  | **Supplementary Table 1. Median typical mean heart doses estimated for 683 women who underwent radiation therapy for breast cancer in the Netherlands between 1970 and 2009 by year of treatment, laterality of cancer and targets irradiated** | | | | | | | | | | | |
| --- | --- | --- | --- | --- | --- | --- | --- | --- | --- | --- | --- | --- |
|  |  |  |  | **Treatment period** | | | | | | | | |
|  |  |  |  | **1970-1986** | |  | **1987-2000** | |  | **2001-2009** | |  |
|  |  |  |  | **No. of** | **Typical mean heart dose** |  | **No. of** | **Typical mean heart dose** |  | **No. of** | **Typical mean heart dose** |  |
|  | **Radiation fields** | |  | **women** | **Median (IQR)** |  | **women** | **Median (IQR)** |  | **women** | **Median (IQR)** |  |
|  |  |  |  |  |  |  |  |  |  |  |  |  |
|  | **All women** | |  | **288** | **8.8 (5.1-15.0)** |  | **257** | **4.8 (2.2-13.4)** |  | **138** | **1.5 (0.3-4.0)** |  |
|  |  |  |  |  |  |  |  |  |  |  |  |  |
|  | **Right IMC (181)** | |  | **109** |  |  |  |  |  |  |  |  |
|  |  | IMC, breast (63) |  | 26 | 15.3 (7.9-16.6) |  | 29 | 13.4 (12.0-16.6) |  | 8 | 9.2 (1.7-9.4) |  |
|  |  | IMC (No chest wall/breast) (90) |  | 77 | 8.9 (5.1-11.0) |  | 12 | 9.9 (8.6-11.1) |  | 1 | 9.4 (9.4-9.4) |  |
|  |  | IMC, chest wall (28) |  | 6 | 12.6 (4.0-13.1) |  | 13 | 10.5 (10.1-12.3) |  | 9 | 1.7 (1.7-9.4) |  |
|  | **Left IMC (190)** | |  | **121** |  |  |  |  |  |  |  |  |
|  |  | IMC, breast (67) |  | 42 | 16.6 (8.3-28.8) |  | 15 | 21.8 (20.9-22.0) |  | 10 | 9.1 (9.0-9.2) |  |
|  |  | IMC (No chest wall/breast) (86) |  | 68 | 12.2 (12.0-16.5) |  | 17 | 16.5 (14.7-18.3) |  | 1 | 16.1 (16.1-16.1) |  |
|  |  | IMC, chest wall (37) |  | 11 | 14.8 (8.0-22.3) |  | 14 | 16.4 (16.4-18.4) |  | 12 | 16.1 (9.0-16.1) |  |
|  | **Right No IMC (136)** | |  | **24** |  |  |  |  |  |  |  |  |
|  |  | Breast only (No IMC) (121) |  | 19 | 0.6 (0.6-0.7) |  | 60 | 0.7 (0.7-0.7) |  | 42 | 0.3 (0.3-0.3) |  |
|  |  | Chest wall only (No IMC) (15) |  | 5 | 2.8 (1.4-2.8) |  | 7 | 2.8 (2.6-2.8) |  | 3 | 0.3 (0.3-0.3) |  |
|  | **Left No IMC (176)** | |  | **34** |  |  |  |  |  |  |  |  |
|  |  | Breast only (No IMC) (154) |  | 26 | 4.3 (4.3-4.8) |  | 79 | 4.8 (4.8-4.8) |  | 49 | 1.5 (1.5-1.5) |  |
|  |  | Chest wall only (No IMC)(22) |  | 8 | 4.0 (3.5-4.6) |  | 11 | 6.3 (5.8-6.3) |  | 3 | 1.5 (1.5-3.9) |  |

Abbreviations: IQR, inter quartile range; IMC, internal mammary chain

| **Supplementary Table 2. Associations between cardiovascular diseases and established cardiovascular risk factors at breast cancer diagnosis, smoking status, and history of cardiovascular diseases in our of hospital-based cohort of 14,645 breast cancer patients** | | | | | | | | | | | | | | | | | | | | |  |  |
| --- | --- | --- | --- | --- | --- | --- | --- | --- | --- | --- | --- | --- | --- | --- | --- | --- | --- | --- | --- | --- | --- | --- |
| **Risk factor** | | | | **Any cardiovascular event** | | | | **Ischemic heart disease*** | | | | **Valvular heart disease** | | | | **Heart failure**^†^ | | |  | |  |  |
| **Multivariable model I**^‡^ | |  | **n/N**^§^ | **HR** | | **(95%CI)** | **n/N**^§^ | **HR** | | **(95%CI)** | **n/N**^§^ | **HR** | | **(95%CI)** | **n/N**^§^ | **HR** | | | **(95%CI)** |  |  |  |
| **Radiation field** | | | |  |  |  | |  |  |  | |  |  |  | |  |  |  | | |  |  |
| Unknown | | | | 79/685 | 1.72 | 1.33-2.23 | | 19/286 | 2.52 | 1.48-4.30 | | 15/728 | 0.98 | 0.54-1.76 | | 24/709 | 2.09 | 1.24-3.51 | | |  |  |
|  | | | |  |  |  | |  |  |  | |  |  |  | |  |  |  | | |  |  |
| **Age at breast cancer diagnosis** | | | |  |  |  | |  |  |  | |  |  |  | |  |  |  | | |  |  |
| <40 years | | | | 172/2,091 | 1.00 | Ref. | | 53/1,544 | 1.00 | Ref. | | 40/2,102 | 1.00 | Ref. | | 29/2,040 | 1.00 | Ref. | | |  |  |
| 40-49 years | | | | 734/5,626 | 1.77 | 1.50-2.09 | | 282/4,102 | 1.98 | 1.47-2.66 | | 204/5,667 | 1.86 | 1.32-2.62 | | 168/5,546 | 1.91 | 1.28-2.84 | | |  |  |
| 50-61 years | | | | 785/4,500 | 3.47 | 2.92-4.11 | | 297/2,936 | 3.73 | 2.76-5.05 | | 207/4,540 | 3.34 | 2.35-4.75 | | 184/4,399 | 3.82 | 2.55-5.73 | | |  |  |
|  | | | |  |  |  | |  |  |  | |  |  |  | |  |  |  | | |  |  |
| **CVD risk factor at breast cancer (excl. smoking)** | | | |  |  |  | |  |  |  | |  |  |  | |  |  |  | | |  |  |
| None present | |  | | 1,517/11,594 | 1.00 | Ref. | | 535/7,939 | 1.00 | Ref. | | 407/11,673 | 1.00 | Ref. | | 329/11,149 | 1.00 | Ref. | | |  |  |
| At least one | |  | | 174/623 | 1.61 | 1.37-1.89 | | 89/501 | 1.62 | 1.29-2.04 | | 44/636 | 1.10 | 0.80-1.52 | | 47/606 | 1.41 | 1.02-1.93 | | |  |  |
|  | |  | |  |  |  | |  |  |  | |  |  |  | |  |  |  | | |  |  |
| **Smoking** | |  | |  |  |  | |  |  |  | |  |  |  | |  |  |  | | |  |  |
| Never | |  | | 502/2,452 | 1.00 | Ref. | | 225/2,336 | 1.00 | Ref. | | 156/2,476 | 1.00 | Ref. | | 110/2,312 | 1.00 | Ref. | | |  |  |
| Ever | |  | | 562/2,765 | 1.38 | 1.22-1.56 | | 248/2,278 | 1.50 | 1.25-1.80 | | 146/2,803 | 1.14 | 0.91-1.44 | | 135/2,662 | 1.50 | 1.16-1.95 | | |  |  |
| Unknown | |  | | 627/7,000 | 0.88 | 0.77-1.00 | | 151/3,826 | 0.83 | 0.67-1.03 | | 149/7,030 | 0.91 | 0.71-1.16 | | 131/6,781 | 0.88 | 0.66-1.18 | | |  |  |
| **History of other CVDs before CVD event of interest** | | | |  |  |  | |  |  |  | |  |  |  | |  |  |  | | |  |  |
| Ischemic heart disease | |  | | n.a. |  |  | | n.a. |  |  | | 90/12,309 | 2.00 | 1.53-2.61 | | 83/11,755 | 2.96 | 2.25-3.88 | | |  |  |
| Valvular heart disease | |  | | n.a. |  |  | | 3/8,440 | 3.78 | 2.08-3.70 | | n.a. | - |  | | 61/11,755 | 3.50 | 2.53-4.85 | | |  |  |
| Heart failure | |  | | n.a. |  |  | | 1/8,440 | 3.23 | 2.35-4.44 | | 67/12,309 | 5.93 | 4.36-8.06 | | n.a. |  |  | | |  |  |
| Other | |  | | n.a. |  |  | | 4/8,440 | 1.48 | 1.14-1.91 | | 80/12,309 | 2.37 | 1.80-3.11 | | 74/11,755 | 2.73 | 2.04-3.65 | | |  |  |

| **Supplementary Table 2. Continued** | | | | | | | | | | | | | |  |
| --- | --- | --- | --- | --- | --- | --- | --- | --- | --- | --- | --- | --- | --- | --- |
| **Risk factor** | | **Any cardiovascular event** | | | **Ischemic heart disease*** | | | **Valvular heart disease** | | | **Heart failure**^†^ | |  |  |
| **Multivariable model II**^\|\|^ |  | **n/N**^§^ | **HR** | **(95%CI)** | **n/N**^§^ | **HR** | **(95%CI)** | **n/N**^§^ | **HR** | **(95%CI)** | **n/N**^§^ | **HR** | **(95%CI)** |  |
| **Radiation target^\|\|^** |  |  |  |  |  |  |  |  |  |  |  |  |  |  |
| Breast only, right-sided (no IMC) |  | 230/2,562 | 1.00 | Ref. | 48/1,676 | 1.00 | Ref. | 51/2,519 | 1.00 | Ref. | 40/2,520 | 1.00 | Ref. |  |
| Chest wall only, right-sided (no IMC^¶^) | | 61/315 | 1.23 | 0.92-1.65 | 23/235 | 1.69 | 1.01-2.80 | 10/349 | 0.50 | 0.25-1.01 | 23/350 | 1.68 | 0.88-2.77 |  |
| IMC, right-sided |  | 193/899 | 1.51 | 1.24-1.85 | 111/741 | 2.82 | 2.00-4.00 | 44/895 | 1.02 | 0.67-1.55 | 49/896 | 1.83 | 1.10-2.71 |  |
| IMC+breast, right-sided |  | 93/597 | 1.36 | 1.06-1.73 | 41/476 | 2.02 | 1.32-3.08 | 33/598 | 1.61 | 1.02-2.52 | 23/597 | 1.74 | 1.08-3.08 |  |
| IMC+chest wall, right-sided |  | 58/308 | 1.70 | 1.27-2.28 | 28/250 | 2.78 | 1.73-4.46 | 20/331 | 1.43 | 0.84-0.42 | 18/331 | 1.69 | 0.92-3.04 |  |
| Breast only, left-sided (no IMC) |  | 272/2,761 | 1.11 | 0.93-1.32 | 70/1,813 | 1.38 | 0.96-1.99 | 56/2,797 | 1.00 | 0.68-1.46 | 41/2,798 | 0.87 | 0.59-1.43 |  |
| Chest wall only, left-sided (no IMC^¶^) | | 71/302 | 1.82 | 1.38-2.38 | 26/216 | 2.42 | 1.49-3.92 | 16/352 | 0.89 | 0.50-1.60 | 20/352 | 1.41 | 0.80-2.74 |  |
| IMC, left-sided |  | 229/1,021 | 1.58 | 1.31-1.91 | 113/867 | 2.28 | 1.62-3.22 | 87/1,014 | 1.88 | 1.31-2.70 | 56/1,013 | 1.60 | 0.98-2.35 |  |
| IMC+breast, left-sided |  | 113/618 | 1.81 | 1.44-2.27 | 48/473 | 2.40 | 1.60-3.60 | 44/623 | 2.33 | 1.54-3.52 | 28/625 | 1.90 | 1.14-3.11 |  |
| IMC+chest wall, left-sided |  | 71/324 | 1.69 | 1.29-2.22 | 28/267 | 1.77 | 1.11-2.86 | 31/365 | 1.92 | 1.21-3.04 | 34/364 | 2.97 | 1.60-4.31 |  |
|  |  |  |  |  |  |  |  |  |  |  |  |  |  |  |
| No radiation therapy |  | 221/1,825 | 1.21 | 1.01-1.46 | 69/1,151 | 1.49 | 1.03-2.16 | 44/1,738 | 0.98 | 0.51-1.16 | 44/1,741 | 1.22 | 0.82-1.99 |  |
| **Chemotherapy^\|\|^** |  |  |  |  |  |  |  |  |  |  |  |  |  |  |
| No chemotherapy |  | 1,258/8,238 | 1.00 | Ref. | 499/6,022 | 1.00 | Ref. | 336/8,296 | 1.00 | Ref. | 274/8,301 | 1.00 | Ref. |  |
| CMF-like regimen |  | 240/1,727 | 1.00 | 0.86-1.16 | 104/1,352 | 1.06 | 0.84-1.32 | 72/1,751 | 1.12 | 0.85-1.47 | 44/1,749 | 1.01 | 0.72-1.42 |  |
| Anthracycline-containing regimen | | 193/2,252 | 1.48 | 1.24-1.75 | 21/1,066 | 0.95 | 0.61-1.52 | 43/2,262 | 1.90 | 1.32-2.75 | 84/2,263 | 4.52 | 3.29-6.22 |  |

Abbreviations: n/N, number of events/number at risk; HR, hazard ratio; CI, confidence interval; Ref., reference category; CVD, cardiovascular disease; n.a., not applicable.

The analyses shown in this table include all diagnoses of cardiovascular disease, e.g. if a patient was diagnosed with ischemic heart disease and then later with valvular heart disease then both are listed. Analyses considering just the first diagnosis of cardiovascular disease are in Supplementary Table 4.

* Because the proportional hazard assumption did not hold for the ischemic heart disease rate after internal mammary chain and chest wall irradiation, results are shown for ten years or more after breast cancer treatment.

^†^ Heart failure included both cardiomyopathy and congestive heart failure; diagnoses I42 and I50 International Classification of Diseases, 10^th^ revision.

^‡^ Hazard ratios estimated using one multivariable model containing radiation fields (right breast, right-sided chest wall, right-sided internal mammary chain field, left breast, left-sided chest wall, left-sided internal mammary chain field, no radiation therapy, unknown radiation fields), chemotherapy (no chemotherapy, CMF-like regimen, anthracycline-containing regimen), age at breast cancer treatment (<40, 40-49, 50-61 years), cardiovascular risk factor at breast cancer diagnosis yes/no (hypertension, hypercholesterolemia, or diabetes), smoking (ever, never, or unknown), and other cardiovascular diseases (time-dependent).

^§^ Analyses included all patients with at least one day cardiovascular follow-up after start of time at risk (n=12.355). Patients with a specific cardiovascular diagnosis before start of time at risk were excluded from analysis with that specific diagnosis as endpoint (n=138 for any cardiovascular event, n=50 for ischemic heart disease, n=18 for valvular heart disease, and n=15 for heart failure). Numbers at risk differs by endpoint due to time-dependency of the treatment variables.

^||^ Hazard ratios estimated using one multivariable model containing radiation fields right breast, right-sided chest wall, right-sided internal mammary chain field, left breast, left-sided chest wall, left-sided internal mammary chain field, no radiation therapy, unknown radiation fields), chemotherapy (no chemotherapy, CMF-like regimen, anthracycline-containing regimen), age at breast cancer treatment (<40, 40-49, 50-61 years), cardiovascular risk factor at breast cancer diagnosis yes/no (hypertension, hypercholesterolemia, or diabetes), smoking (ever, never, or unknown), and other cardiovascular diseases (time-dependent).

| **Supplementary Table 3. Within cohort comparison of cardiovascular disease risks after breast cancer by treatment in patients age fifty or younger at breast cancer diagnosis** | | | | | | | | | | | | | | | | |  |  |  |  |  |  |
| --- | --- | --- | --- | --- | --- | --- | --- | --- | --- | --- | --- | --- | --- | --- | --- | --- | --- | --- | --- | --- | --- | --- |
|  | **Any cardiovascular event** | | | **Ischemic heart disease ≥10 years after breast cancer treatment*** | | | **Valvular heart disease** | | | | | **Heart failure** | | | | |  |  |  |  |  |  |
| **Multivariable model**^†^ | **n/N** | **HR** | **(95%CI)** | **n/N** | **HR** | **(95%CI)** | **n/N** | | **HR** | | **(95%CI)** | **n/N** | | **HR** | | **(95%CI)** | | |  |  |  |  |
| **Radiation field** |  |  |  |  |  |  |  | |  | |  |  | |  | |  | | |  |  |  |  |
| Breast only, right-sided (no IMC) | 134/1,712 | 1.00 | Ref. | 43/1,719 | 1.00 | Ref. | 30/1,673 | | 1.00 | | Ref. | 22/1,648 | | 1.00 | | Ref. | | |  |  |  |  |
| Chest wall only, right-sided (no IMC) | 40/221 | 1.30 | 0.90-1.88 | 15/221 | 2.16 | 1.10-4.22 | 6/241 | | - | |  | 18/226 | | 2.02 | | 1.04-3.93 | | |  | |  | |
| IMC, right-sided | 117/607 | 1.66 | 1.28-2.14 | 62/609 | 3.40 | 2.09-5.52 | 27/605 | | 1.15 | | 0.67-1.96 | 26/586 | | 1.78 | | 0.98-3.21 | | |  | |  | |
| IMC+breast, right-sided | 69/451 | 1.65 | 1.23-2.22 | 34/451 | 3.32 | 1.94-5.67 | 26/448 | | 1.92 | | 1.12-3.31 | 12/435 | | 1.57 | | 0.77-3.20 | | |  | |  | |
| IMC+chest wall, right-sided | 35/206 | 180 | 1.23-2.63 | 14/206 | 2.85 | 1.46-5.58 | 14,223 | | 1.44 | | 0.75-2.76 | 9/202 | | 1.35 | | 0.61-2.98 | | |  | |  | |
|  |  |  |  |  |  |  |  | |  | |  |  | |  | |  | | |  | |  | |
| Breast only, left-sided (no IMC) | 149/1,830 | 1.06 | 0.84-1.34 | 59/1,839 | 1.70 | 1.03-2.80 | 26/1,854 | | 0.78 | | 0.46-1.94 | 26/1,830 | | 1.02 | | 0.58-1.80 | | |  | |  | |
| Chest wall only, left-sided (no IMC) | 46/222 | 1.84 | 1.31-2.59 | 17/223 | 2.46 | 1.26-4.78 | 11/265 | | 0.94 | | 0.46-1.94 | 10/235 | | 1.25 | | 0.57-2.74 | | |  | |  | |
| IMC, left-sided | 148/683 | 1.82 | 1.43-2.32 | 83/689 | 3.49 | 2.18-5.57 | 53/678 | | 1.98 | | 1.25-3.15 | 33/667 | | 1.68 | | 0.95-2.95 | | |  | |  | |
| IMC+breast, left-sided | 76/445 | 1.88 | 1.41-2.51 | 32/449 | 3.18 | 1.85-5.46 | 31/449 | | 2.47 | | 1.48-4.14 | 15/442 | | 1.70 | | 0.87-3.33 | | |  | |  | |
| IMC+chest wall, left-sided | 33/208 | 1.37 | 0.93-2.02 | 12/209 | 1.69 | 0.82-3.48 | 17/235 | | 1.87 | | 1.01-3.45 | 15/217 | | 2.34 | | 1.19-4.61 | | |  | |  | |
|  |  |  |  |  |  |  |  | |  | |  |  | |  | |  | | |  | |  | |
| No radiation therapy | 116/1,166 | 1.18 | 0.94-1.56 | 48/1,171 | 2.24 | 1.35-3.73 | 19/1,116 | | 0.57 | | 0.31-1.02 | 18/1,107 | | 1.01 | | 0.80-3.49 | | |  | |  | |
| **Chemotherapy** |  |  |  |  |  |  |  | |  | |  |  | |  | |  | | |  |  |  |  |
| No chemotherapy | 697/5,110 | 1.00 | Ref. | 323/5,131 | 1.00 | Ref. | 189/5,144 | | 1.00 | | Ref. | 130/4,978 | | 1.00 | | Ref. | | |  |  |  |  |
| CMF-like regimen | 187/1,524 | 0.95 | 0.80-1.13 | 82/1,537 | 0.94 | 0.71-1.23 | 52/1,543 | | 0.99 | | 0.71-1.37 | 35/1,512 | | 1.14 | | 0.77-1.70 | | |  | |  | |
| Anthracycline-containing regimen | 107/1,527 | 1.49 | 1.19-1.88 | 25/1,530 | 0.92 | 0.50-1.68 | 26/1,532 | 2.41 | | 1.48-3.93 | | 50/1,532 | 5.23 | | 3.41-8.01 | | |  | |  | |  |

Abbreviations: IQR, interquartile range; n/N, number of events/number at risk; HR, hazard ratio; CI, confidence interval; IMC, internal mammary chain; Ref., reference category; CVD, cardiovascular disease; n.a., not applicable; RT, radiation therapy.

The analyses shown in this table include all diagnoses of cardiovascular disease, e.g. if a patient was diagnosed with ischemic heart disease and then later with valvular heart disease then both are listed.

*Because the proportional hazard assumption did not hold for the ischemic heart disease rate after internal mammary chain and chest wall irradiation, results are shown for ten years or more after breast cancer treatment. No increased ischemic heart disease risks were seen in the time period before ten years after treatment.

^†^ Hazard ratios estimated using one multivariable model containing radiation fields (right breast, right-sided chest wall, right-sided internal mammary chain field, left breast, left-sided chest wall, left-sided internal mammary chain field, no radiation therapy, unknown radiation fields), chemotherapy (no chemotherapy, CMF-like regimen, anthracycline-containing regimen), age at breast cancer treatment (<40, 40-49, 50-61 years), cardiovascular risk factor at breast cancer diagnosis yes/no (hypertension, hypercholesterolemia, or diabetes), smoking (ever, never, or unknown), and other cardiovascular diseases (time-dependent). Analyses included all patients with at least one day of cardiovascular follow-up after start of time at risk (n=8.161). Patients with a specific cardiovascular diagnosis before start of time at risk were excluded from analysis with that specific diagnosis as endpoint (n=88 for any cardiovascular event, n=29 for ischemic heart disease, n=11 for valvular heart disease, and n=23 for heart failure).

| **Supplementary Table 4.** **Within cohort comparison of cardiovascular disease rates after breast cancer by treatment, taking into account each patient’s first cardiovascular diagnosis only** | | | | | | | | | | | | | | | | | | | |  |  |  |  |  |
| --- | --- | --- | --- | --- | --- | --- | --- | --- | --- | --- | --- | --- | --- | --- | --- | --- | --- | --- | --- | --- | --- | --- | --- | --- |
|  | **Any cardiovascular event** | | | **Ischemic heart disease ≥10 years after breast cancer treatment*** | | | | **Valvular heart disease** | | | | | | **Heart failure**^†^ | | | | | |  |  |  |  |  |
| **Multivariable model**^‡^ | **n/N**^§^ | **HR** | **(95%CI)** | **n/N**^§^ | **HR** | **(95%CI)** | **n/N**^§^ | | | **HR** | | **(95%CI)** | **n/N** | | | **HR** | | **(95%CI)** |  |  |  |  |  |  |
| **Radiation field^\|\|^** |  |  |  |  |  |  |  | | |  | |  |  | | |  | |  | | |  |  |  |  |
| Breast only, right-sided (no IMC) | 192/2,476 | 1.00 | Ref. | 30/1,432 | 1.00 | Ref. | 29/2,476 | | | 1.00 | | Ref. | 18/2,476 | | | 1.00 | | Ref. | | |  |  |  |  |
| Chest wall only, right-sided (no IMC) | 44/261 | 1.17 | 0.84-1.65 | 16/190 | 1.90 | 1.02-3.53 | 2/261 | | | 0.29 | | 0.07-1.21 | 8/261 | | | 1.99 | | 0.83-4.80 | | |  | |  | |
| IMC, right-sided | 147/811 | 1.46 | 1.17-1.83 | 72/631 | 3.07 | 1.99-4.76 | 11/811 | | | 0.59 | | 0.29-1.20 | 17/811 | | | 1.82 | | 0.92-3.64 | | |  | |  | |
| IMC+breast, right-sided | 78/549 | 1.44 | 1.10-1.89 | 25/423 | 2.28 | 1.33-3.90 | 17/549 | | | 1.76 | | 0.95-3.26 | 10/549 | | | 1.91 | | 0.87-4.19 | | |  | |  | |
| IMC+chest wall, right-sided | 45/276 | 1.68 | 1.21-2.33 | 16/216 | 2.78 | 1.50-5.13 | 8/276 | | | 1.73 | | 0.77-3.85 | 8/276 | | | 2.40 | | 1.02-5.63 | | |  | |  | |
| Breast only, left-sided (no IMC) | 218/2,652 | 1.07 | 0.88-1.30 | 43/1,562 | 1.34 | 0.84-2.14 | 32/2,652 | | | 1.03 | | 0.62-1.70 | 22/2,652 | | | 1.17 | | 0.63-2.19 | | |  | |  | |
| Chest wall only, left-sided (no IMC) | 41/234 | 1.50 | 1.06-2.12 | 15/160 | 2.48 | 1.31-4.67 | 2/234 | | | 0.20 | | 0.03-1.48 | 3/234 | | | 0.95 | | 0.27-3.33 | | |  | |  | |
| IMC, left-sided | 171/914 | 1.52 | 1.23-1.88 | 65/729 | 2.34 | 1.51-3.64 | 44/914 | | | 2.05 | | 1.25-3.37 | 16/914 | | | 1.48 | | 0.74-2.96 | | |  | |  | |
| IMC+breast, left-sided | 72/578 | 1.44 | 1.09-1.90 | 23/399 | 2.40 | 1.38-4.16 | 16/578 | | | 1.77 | | 0.94-3.34 | 6/578 | | | 1.22 | | 0.48-3.12 | | |  | |  | |
| IMC+chest wall, left-sided | 51/273 | 1.47 | 1.07-2.02 | 15/215 | 1.88 | 1.00-3.52 | 13/273 | | | 2.17 | | 1.10-4.28 | 7/273 | | | 1.76 | | 0.72-4.31 | | |  | |  | |
| No radiation therapy | 175/1,607 | 1.21 | 0.98-1.49 | 41/966 | 1.45 | 0.90-2.34 | 19/1,607 | | | 0.76 | | 0.42-1.36 | 26/1,607 | | | 1.92 | | 1.04-3.53 | | |  | |  | |
| **Chemotherapy^\|\|^** |  |  |  |  |  |  |  | | |  | |  |  | | |  | |  | | |  |  |  |  |
| No chemotherapy | 962/7,504 | 1.00 | Ref. | 305/5,049 | 1.00 | Ref. | 145/7,504 | | | 1.00 | | Ref. | 97/7,504 | | | 1.00 | | Ref. | | |  |  |  |  |
| CMF-like regimen | 172/1,614 | 0.95 | 0.80-1.13 | 53/1,136 | 0.83 | 0.61-1.12 | 36/1,614 | | | 1.28 | | 0.86-1.89 | 15/1,614 | | | 0.95 | | 0.53-1.69 | | |  | |  | |
| Anthracycline-containing regimen | 162/2,151 | 1.44 | 1.20-1.74 | 14/969 | 0.94 | 0.54-1.65 | 15/2,151 | | 1.11 | | 0.62-2.00 | | 38/2,151 | | 3.93 | | 2.49-6.22 | | |  | |  | |  |
| **Multivariable model II**^‡^ |  |  |  |  |  |  |  | |  | |  | |  | |  | |  | | |  | |  | |  |
| **Radiation target^\|\|^** |  |  |  |  |  |  |  | |  | |  | |  | |  | |  | | |  | |  | |  |
| Breast, right-sided (no IMC) | 192/2,476 | 1.00 | Ref. | 30/1,432 | 1.00 | Ref. | 29/2,476 | | 1.00 | | Ref. | | 18/2,476 | | 1.00 | | Ref. | | |  | |  | |  |
| Chest wall, right-sided (no IMC^¶^) | 44/261 | 1.17 | 0.84-1.65 | 16/190 | 1.90 | 1.02-3.53 | 2/261 | | 0.29 | | 0.07-1.22 | | 8/261 | | 1.98 | | 0.82-4.77 | | |  | |  | |  |
| IMC, right-sided (+/- breast/chest wall) | 271/1,636 | 1.49 | 1.23-1.81 | 113/1,270 | 2.80 | 1.85-4.23 | 36/1,636 | | 1.10 | | 0.66-1.83 | | 35/1,636 | | 1.96 | | 1.09-3.54 | | |  | |  | |  |
| Breast, left-sided (no IMC) | 218/2,652 | 1.07 | 0.88-1.30 | 43/1,562 | 1.34 | 0.84-2.13 | 32/2,652 | | 1.03 | | 0.62-1.71 | | 22/2,652 | | 1.17 | | 0.63-2.19 | | |  | |  | |  |
| Chest wall, left-sided (no IMC^¶^) | 41/234 | 1.49 | 1.05-2.12 | 15/160 | 2.46 | 1.30-4.63 | 2/234 | | 0.20 | | 0.03-1.50 | | 3/234 | | 0.94 | | 0.27-3.31 | | |  | |  | |  |
| IMC, left-sided (+/- breast/chest wall) | 294/1,765 | 1.49 | 1.23-1.81 | 103/1,343 | 2.27 | 1.50-3.44 | 73/1,765 | | 2.00 | | 1.27-3.17 | | 29/1,765 | | 1.48 | | 0.80-2.71 | | |  | |  | |  |
| No radiation therapy | 175/1,607 | 1.21 | 0.98-1.49 | 41/966 | 1.44 | 0.90-2.32 | 19/1,607 | | 0.77 | | 0.43-1.38 | | 26/1,607 | | 1.91 | | 1.04-3.52 | | |  | |  | |  |
| **Chemotherapy^\|\|^** |  |  |  |  |  |  |  | |  | |  | |  | |  | |  | | |  | |  | |  |
| No chemotherapy | 962/7,504 | 1.00 | Ref. | 305/5,049 | 1.00 | Ref. | 145/7,504 | | 1.00 | | Ref. | | 97/7,504 | | 1.00 | | Ref. | | |  | |  | |  |
| CMF-like regimen | 172/1,614 | 0.94 | 0.79-1.12 | 53/1,136 | 0.83 | 0.61-1.12 | 36/1,614 | | 1.26 | | 0.85-1.85 | | 15/1,614 | | 0.93 | | 0.52-1.65 | | |  | |  | |  |
| Anthracycline-based regimen | 162/2,151 | 1.48 | 1.20-1.82 | 14/969 | 1.04 | 0.56-1.91 | 15/2,151 | | 0.95 | | 0.49-1.82 | | 38/2,151 | | 4.17 | | 2.50-6.94 | | |  | |  | |  |
| **Endocrine therapy** |  |  |  |  |  |  |  | |  | |  | |  | |  | |  | | |  | |  | |  |
| No endocrine therapy | 1,156/9,374 | 1.00 | Ref. | 359/6,320 | 1.00 | Ref. | 175/9,374 | | 1.00 | | Ref. | | 125/9,374 | | 1.00 | | Ref. | | |  | |  | |  |
| Endocrine therapy | 140/1,895 | 0.96 | 0.78-1.20 | 13/834 | 0.77 | 0.41-1.42 | 21/1,895 | | 1.50 | | 0.86-2.61 | | 25/1,895 | | 0.92 | | 0.54-1.59 | | |  | |  | |  |
| **Summary model** |  |  |  |  |  |  |  | |  | |  | |  | |  | |  | | |  | |  | |  |
| Breast, right-sided (no IMC) | 192/2,476 | 1.00 | Ref. | 30/1,432 | 1.00 | Ref. | 29/2,476 | | 1.00 | | Ref. | | 18/2,476 | | 1.00 | | Ref. | | |  | |  | |  |
| IMC (left- or right-sided, +/- breast/chest wall) | 565/3,401 | 1.49 | 1.25-1.77 | 216/2,613 | 2.51 | 1.70-3.72 | 109/3,401 | | 1.57 | | 1.02-2.44 | | 64/3,401 | | 1.71 | | 0.99-2.94 | | |  | |  | |  |

Abbreviations: n/N, number of events/number at risk; HR, hazard ratio; CI, confidence interval; IMC, internal mammary chain; Ref., reference category.

The analyses shown in this table include first cardiovascular disease event only, e.g. if a patient was diagnosed with ischemic heart disease and then later with valvular heart disease only ischemic heart disease is listed. If ischemic heart disease was diagnosed on the same day as valvular heart disease or heart failure, only ischemic heart disease is listed. In case of a valvular heart disease and heart failure diagnosis on the same day, neither is listed.

* Because the proportional hazard assumption did not hold for the ischemic heart disease rate after internal mammary chain and chest wall irradiation, results are shown here for ten or more years after breast cancer treatment. No increased ischemic heart disease rates were seen in the period less than ten years after treatment. These results are presented in Supplementary Table 5.

^†^ Heart failure included both cardiomyopathy and congestive heart failure; diagnoses I42 and I50 International Classification of Diseases, 10^th^ revision.

^‡^ Hazard ratios estimated using one multivariable model containing radiation fields (right breast, right-sided chest wall, right-sided internal mammary chain field, left breast, left-sided chest wall, left-sided internal mammary chain field, no radiation therapy, unknown radiation fields), chemotherapy (no chemotherapy, CMF-like regimen, anthracycline-containing regimen), cardiovascular risk factor at breast cancer diagnosis yes/no (hypertension, hypercholesterolemia, or diabetes), and smoking (ever, never, or unknown). The analyses were stratified for age at breast cancer diagnosis ((<40, 40-49, 50-61 years). Patients were censored at diagnosis of any recurrence, contralateral breast cancer or other subsequent malignancy.

^§^ Analyses included all patients with no recurrence, contralateral breast cancer or other subsequent malignancy before start of time at risk, and with at least one day of cardiovascular follow-up (n=11.269).

^||^ Mutually exclusive treatment categories, taking into account primary treatment, as well as treatment for (loco)regional recurrences and second breast cancers.

^¶^ For some women who were treated with direct electron chest wall fields the internal mammary chain received a therapeutic dose.

| **Supplementary Table 5. Within cohort comparison of ischemic heart disease rates in the first ten years after breast cancer by treatment** | | | | | | | |  |  |  |
| --- | --- | --- | --- | --- | --- | --- | --- | --- | --- | --- |
|  | | | **Ischemic heart disease*** | | | | |  |  |  |
| **Multivariable model**^†^ | | **n/N** | | **HR** | | **(95%CI)** | |  |  |  |
| **Radiation target^‡^** | | |  |  | |  | |  | |  |
| Breast only, right-sided (no IMC) | |  | 36/886 | 1.00 | | Ref. | |  | |  |
| Chest wall only, right-sided (no IMC^§^) | | | 2/73 | - | |  | |  | |  |
| IMC, right-sided | |  | 10/332 | 0.83 | | 0.40-1.72 | |  |  |  |
|  | |  |  |  | |  | |  | |  |
| Breast only, left-sided (no IMC) | |  | 35/953 | 0.92 | | 0.58-1.46 | |  | |  |
| Chest wall, left-sided (no IMC^§^) | | | 4/77 | - | |  | |  | |  |
| IMC, left-sided | |  | 15/360 | 1.14 | | 0.61-2.15 | |  | |  |
|  | |  |  |  | |  | |  |  |  |
| No radiation therapy | |  | 16/611 | 0.91 | | 0.50-1.64 | |  |  |  |
| **Chemotherapy^‡^** | |  |  |  | |  | |  |  |  |
| No chemotherapy | |  | 89/2,164 | 1.00 | | Ref. | |  |  |  |
| CMF-like regimen | |  | 13/379 | 0.93 | | 0.49-1.76 | |  |  |  |
| Anthracycline-containing regimen | | | 28/1,151 | 0.87 | | 0.55-1.38 | |  |  |  |

Abbreviations: IQR, interquartile range; n/N, number of events/number at risk; HR, hazard ratio; CI, confidence interval; IMC, internal mammary chain; Ref., reference category; CVD, cardiovascular disease; n.a., not applicable; RT, radiation therapy.

* Because the proportional hazard assumption did not hold for the ischemic heart disease rate after internal mammary chain and chest wall irradiation, results are shown here for the first ten years after breast cancer treatment. The results for ten or more years after treatment are presented in table 3 in the paper.

^†^ Hazard ratios estimated using one multivariable model containing radiation fields (right breast, right-sided chest wall, right-sided internal mammary chain field, left breast, left-sided chest wall, left-sided internal mammary chain field, no radiation therapy, unknown radiation fields), chemotherapy (no chemotherapy, CMF-like regimen, anthracycline-containing regimen), age at breast cancer treatment (<40, 40-49, 50-61 years), cardiovascular risk factor at breast cancer diagnosis yes/no (hypertension, hypercholesterolemia, or diabetes), smoking (ever, never, or unknown), and other cardiovascular diseases (time-dependent).

| **Supplemental table 6. Joint effects analyses of cardiovascular risk factors and smoking with radiotherapy** | | | | | | | | | | | | | |  |
| --- | --- | --- | --- | --- | --- | --- | --- | --- | --- | --- | --- | --- | --- | --- |
|  | | **Any cardiovascular event** | | | **Ischemic heart disease ≥10 years after breast cancer treatment*** | | | **Valvular heart disease** | | | **Heart failure**^†^ | |  |  |
| **Joint effects of CVD risk factors at BC and RT**^\|\|,††^ | | **n/N**^§^ | **HR** | **(95%CI)** | **n/N**^§^ | **HR** | **(95%CI)** | **n/N**^§^ | **HR** | **(95%CI)** | **n/N**^§^ | **HR** | **(95%CI)** |  |
| Breast RT (no IMC), no CVD risk factors |  | 461/5,106 | 1.00 | Ref. | 104/3,359 | 1.00 | Ref. | 100/5,098 | 1.00 | Ref. | 71/5,039 | 1.00 | Ref. |  |
| IMC RT, no CVD risk factors |  | 667/3,517 | 1.52 | 1.34-1.73 | 314/2,861 | 1.89 | 1.42-2.53 | 229/3,567 | 1.77 | 1.67-2.28 | 167/3,429 | 1.96 | 1.47-2.63 |  |
| Breast RT (no IMC), ≥ 1 CVD risk factors | | 41/217 | 1.84 | 1.32-2.56 | 14/146 | 2.45 | 1.23-4.91 | 7/218 | 1.36 | 0.63-2.93 | 8/214 | 2.24 | 1.07-4.66 |  |
| IMC RT, ≥ 1 CVD risk factors |  | 90/250 | 2.25 | 1.78-2.85 | 56/238 | 2.61 | 1.63-4.17 | 31/259 | 2.02 | 1.31-3.12 | 29/247 | 3.15 | 2.02-4.91 |  |
| *Test for departure from additivity/multiplicativity* | |  |  | p=0.89/0.29 |  |  | p=0.87/0.73 |  |  | p=0.98/0.77 |  |  | p=0.79/0.58 |  |
| **Joint effects of smoking and RT**^\|\|,‡‡^ |  |  |  |  |  |  |  |  |  |  |  |  |  |  |
| Breast RT (no IMC), never smoking |  | 95/754 | 1.00 | Ref. | 30/714 | 1.00 | Ref. | 21/756 | 1.00 | Ref. | 14/724 | 1.00 | Ref. |  |
| IMC RT, never smoking |  | 284/1,163 | 1.46 | 1.26-1.70 | 141/1,135 | 2.03 | 1.43-2.87 | 112/1,177 | 1.90 | 1.41-2.56 | 69/1,114 | 1.78 | 1.26-2.51 |  |
| Breast RT (no IMC), ever smoking |  | 158/1,209 | 1.30 | 1.05-1.60 | 46/938 | 1.92 | 1.22-3.03 | 36/1,203 | 1.36 | 0.87-2.11 | 25/1,181 | 1.28 | 0.77-2.14 |  |
| IMC RT, ever smoking |  | 285/988 | 1.98 | 1.64-2.37 | 157/931 | 2.87 | 1.92-4.30 | 87/1,017 | 2.01 | 1.40-2.90 | 80/969 | 2.67 | 1.79-3.99 |  |
| *Test for departure from additivity/multiplicativity* | |  |  | p=0.28/0.99 |  |  | p=0.89/0.16 |  |  | p=0.76/0.56 |  |  | p=0.20/0.81 |  |

Abbreviations: n/N, number of events/number at risk; HR, hazard ratio; CI, confidence interval; IMC, internal mammary chain; Ref., reference category; CVD, cardiovascular disease; n.a., not applicable; RT, radiation therapy.

The analyses shown in this table include all diagnoses of cardiovascular disease, e.g. if a patient was diagnosed with ischemic heart disease and then later with valvular heart disease then both are listed.

* Because the proportional hazard assumption did not hold for the IHD rate after IMC and chest wall irradiation, results are shown here for ten or more years after breast cancer treatment. No increased ischemic heart disease rates were seen in the period less than ten years after treatment. These results are presented in Supplementary Table 5.

^†^ Heart failure included both cardiomyopathy and congestive heart failure; diagnoses I42 and I50 International Classification of Diseases, 10^th^ revision.

^§^ Analyses included all patients with at least one day of cardiovascular follow-up after start of time at risk (n=12.355). Patients with a specific cardiovascular diagnosis before start of time at risk were excluded from analysis with that specific diagnosis as endpoint (n=138 for any cardiovascular event, n=50 for ischemic heart disease, n=18 for valvular heart disease, and n=36 for heart failure). Numbers at risk differs by endpoint due to time-dependency of the treatment variables.

^||^ Mutually exclusive treatment categories, taking into account primary treatment, as well as treatment for (loco)regional recurrences and second breast cancers.

^††^ Hazard ratios estimated using one multivariable model containing one variable for the joint effect of radiation therapy and the cardiovascular risk factors hypertension, hypercholesterolemia and diabetes (breast irradiation without cardiovascular risk factors, internal mammary chain irradiation without cardiovascular risk factors, breast irradiation with cardiovascular risk factors, internal mammary chain irradiation with cardiovascular risk factors), age at breast cancer (<40, 40-50, 50-61 years), chemotherapy (no, CMF-like, or anthracycline-based chemotherapy), smoking (ever, never, or unknown), and other cardiovascular diseases (time-dependent). Patients not irradiated to either the breast or internal mammary chain were excluded from these analyses.

^‡‡^ Hazard ratios estimated using one multivariable model containing one variable for the joint effect of radiation therapy and smoking (breast irradiation and never smoking, internal mammary chain irradiation and never smoking, breast irradiation and ever smoking, internal mammary chain irradiation and ever smoking), age at breast cancer (<40, 40-50, 50-61 years), chemotherapy (no, CMF-like, or anthracycline-based chemotherapy), cardiovascular risk factor at breast cancer diagnosis yes/no (hypertension, hypercholesterolemia or diabetes), and other cardiovascular diseases (time-dependent). Patients not irradiated to either the breast or internal mammary chain were excluded from these analyses.

| **Supplementary Table 7. Association between cardiovascular risk factors, history of cardiovascular disease and socioeconomic status with breast cancer treatment** | | | | | | |  |  |  |
| --- | --- | --- | --- | --- | --- | --- | --- | --- | --- |
| **Internal mammary chain irradiation** | | | **No** | **Yes** | | **p** |  |  |  |
| **Cardiovascular risk factor** | |  | | |  |  |  |  | |
| No |  | 97.1% | | | 97.3% |  |  |  | |
| Yes | | 2.9% | | | 2.8% | 0.73 |  |  | |
| **History of cardiovascular disease** | |  | | |  |  |  |  | |
| No | | 98.5% | | | 99.0% |  |  |  | |
| Yes |  | 1.6% | | | 1.0% | 0.31 |  |  | |
| **Socioeconomic status** |  |  | | |  |  |  |  |  |
| Below average |  | 10.9% | | | 9.7% |  |  |  |  |
| Average |  | 61.4% | | | 61.8% |  |  |  |  |
| Above average | | 27.7% | | | 28.4% | 0.05 |  |  |  |
| **Anthracycline-based chemotherapy** | | **No** | | | **Yes** | **p** |  |  |  |
| **Cardiovascular risk factor** | |  | | |  |  |  |  |  |
| No | | 97.0% | | | 97.6% |  |  | |  |
| Yes | | 3.0% | | | 2.4% | 0.12 |  |  |  |
| **History of cardiovascular disease** | |  | | |  |  |  | |  |
| No | | 98.6% | | | 98.6% |  |  |  |  |
| Yes | | 1.5% | | | 1.4% | 0.90 |  | |  |
| **Socioeconomic status** | |  | | |  |  |  | |  |
| Below average | | 9.0% | | | 8.8% |  |  | |  |
| Average | | 60.8% | | | 60.5% |  |  | |  |
| Above average | | 30.2% | | | 30.7% | 0.54 |  |  |  |

Cardiovascular risk factors and history of cardiovascular disease at time of breast cancer diagnosis were considered. Cardiovascular risk factors included hypertension, diabetes, and hypercholesterolemia. Socioeconomic status was assessed using patients’ postal code to calculate the area-level socioeconomic status of each patient. Analyses included patients diagnosed between 1987 and 2009 to assure uniform risk factor assessment for all patients. P-values were calculated using Chi-square tests.

**Supplementary Figure 1. Cumulative risk of cardiovascular diseases in patients diagnosed during 1987-1999, aged 50-61 years at breast cancer diagnosis and not treated with chemotherapy, by internal mammary chain irradiation and cardiovascular disease risk factors (including smoking) at breast cancer diagnosis**

The analyses of ischemic heart disease, valvular heart disease, and heart failure shown in this figure include all diagnoses of cardiovascular disease, e.g. if a patient was diagnosed with ischemic heart disease and then later with valvular heart disease then both events are counted. Patients with a specific cardiovascular diagnosis before start of time at risk were excluded from analysis with that specific diagnosis as endpoint.

Abbreviations: IMC, internal mammary chain; CVD, cardiovascular disease; IHD, ischemic heart disease; VHD, valvular heart disease; HF, heart failure.

**
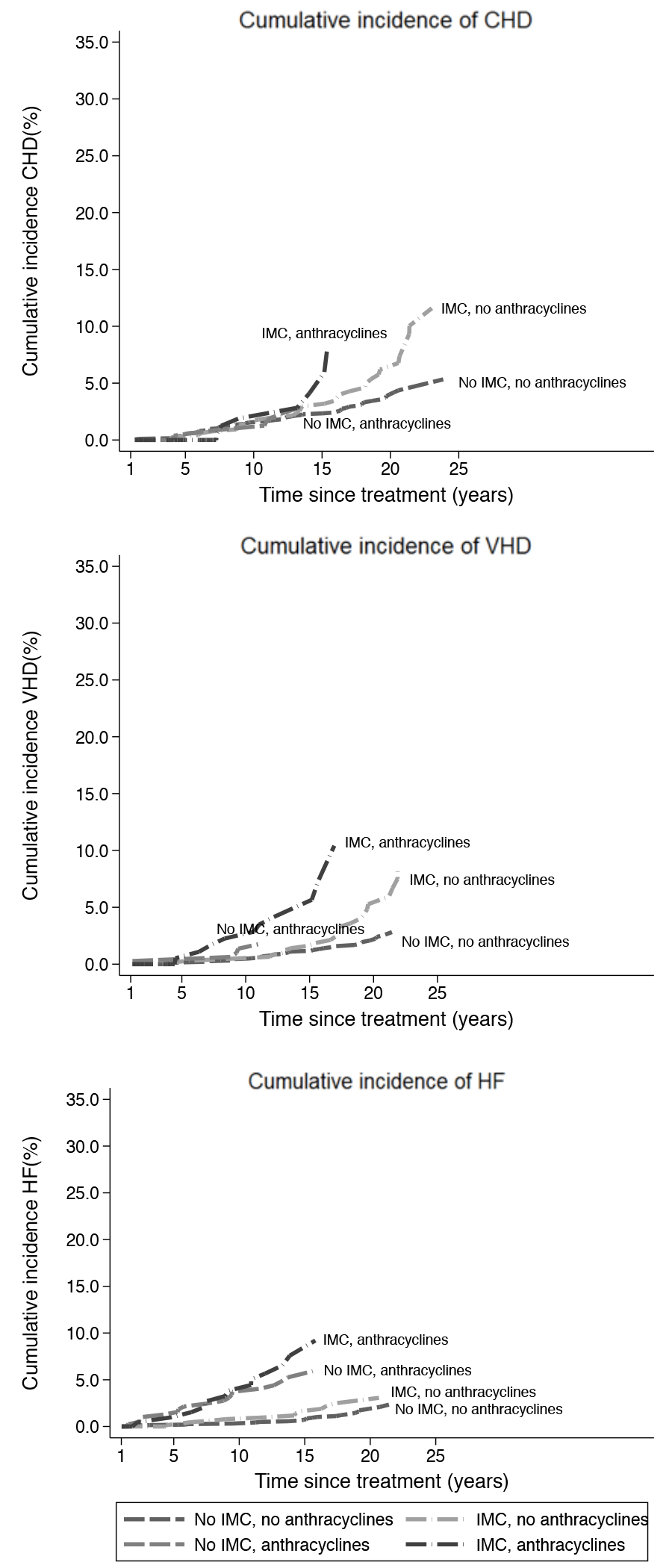
**

**Supplementary Figure 2. Cumulative risk of cardiovascular diseases in patients diagnosed during 1987-1999 and aged 50 years or younger at breast cancer diagnosis, by internal mammary chain irradiation and anthracycline-containing chemotherapy**

Abbreviations: IMC, internal mammary chain; CVD, cardiovascular disease; IHD, ischemic heart disease; VHD, valvular heart disease; HF, heart failure.

The analyses of ischemic heart disease, valvular heart disease, and heart failure shown in this figure include all diagnoses of cardiovascular disease, e.g. if a patient was diagnosed with ischemic heart disease and then later with valvular heart disease then both events are counted. Patients with a specific cardiovascular diagnosis before start of time at risk were excluded from analysis with that specific diagnosis as endpoint.

The cumulative incidences by internal mammary chain irradiation and anthracycline-based chemotherapy Is not shown for patients older than 50 years at breast cancer diagnosis because relatively few patients in this age category were treated with anthracycline-based chemotherapy.

**
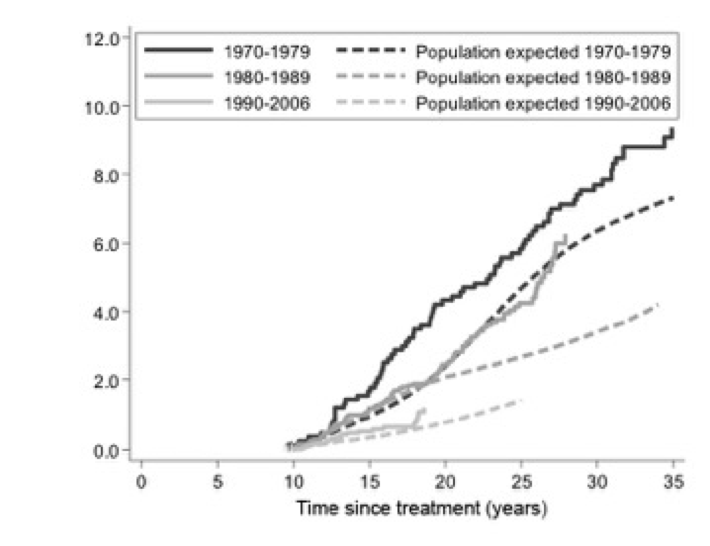
**

**Supplementary Figure 3. Cumulative risk of myocardial infarction in ten-year breast cancer survivors aged 50 years or younger at breast cancer diagnosis, by treatment period, and the expected risk based on age-, sex-, and calendar year- specific myocardial infarction incidence in the general population**

Patients diagnosed with myocardial infarction before breast cancer or within ten years after breast cancer diagnosis were excluded from the analysis
